# Identification of candidate genes controlling cold tolerance at the early seedling stage from Dongxiang wild rice by QTL mapping, BSA-Seq and RNA-Seq

**DOI:** 10.1186/s12870-024-05369-x

**Published:** 2024-07-09

**Authors:** Shiqi Zhou, Ting Wu, Xia Li, Shilin Wang, Biaolin Hu

**Affiliations:** Rice Research Institute, Jiangxi Academy of Agricultural Sciences, No. 602 Nanlian Road, Qingyunpu District, Nanchang, 330000 China

**Keywords:** Dongxiang wild rice, Cold tolerance, RNA-seq, BSA-seq, Gene interaction/coexpression network

## Abstract

**Background:**

The cold tolerance of rice is closely related to its production and geographic distribution. The identification of cold tolerance-related genes is of important significance for developing cold-tolerant rice. Dongxiang wild rice (*Oryza rufipogon* Griff.) (DXWR) is well-adapted to the cold climate of northernmost-latitude habitats ever found in the world, and is one of the most valuable rice germplasms for cold tolerance improvement.

**Results:**

Transcriptome analysis revealed genes differentially expressed between Xieqingzao B (XB; a cold sensitive variety) and 19H19 (derived from an interspecific cross between DXWR and XB) in the room temperature (RT), low temperature (LT), and recovery treatments. The results demonstrated that chloroplast genes might be involved in the regulation of cold tolerance in rice. A high-resolution SNP genetic map was constructed using 120 BC_5_F_2_ lines derived from a cross between 19H19 and XB based on the genotyping-by-sequencing (GBS) technique. Two quantitative trait loci (QTLs) for cold tolerance at the early seedling stage (CTS), *qCTS12* and *qCTS8*, were detected. Moreover, a total of 112 candidate genes associated with cold tolerance were identified based on bulked segregant analysis sequencing (BSA-seq). These candidate genes were divided into eight functional categories, and the expression trend of candidate genes related to ‘oxidation-reduction process’ and ‘response to stress’ differed between XB and 19H19 in the RT, LT and recovery treatments. Among these candidate genes, the expression level of *LOC_Os12g18729* in 19H19 (related to ‘response to stress’) decreased in the LT treatment but restored and enhanced during the recovery treatment whereas the expression level of *LOC_Os12g18729* in XB declined during recovery treatment. Additionally, XB contained a 42-bp deletion in the third exon of *LOC_Os12g18729*, and the genotype of BC_5_F_2_ individuals with a survival percentage (SP) lower than 15% was consistent with that of XB. Weighted gene coexpression network analysis (WGCNA) and modular regulatory network learning with per gene information (MERLIN) algorithm revealed a gene interaction/coexpression network regulating cold tolerance in rice. In the network, differentially expressed genes (DEGs) related to ‘oxidation-reduction process’, ‘response to stress’ and ‘protein phosphorylation’ interacted with *LOC_Os12g18729*. Moreover, the knockout mutant of *LOC_Os12g18729* decreased cold tolerance in early rice seedling stage signifcantly compared with that of wild type.

**Conclusions:**

In general, study of the genetic basis of cold tolerance of rice is important for the development of cold-tolerant rice varieties. In the present study, QTL mapping, BSA-seq and RNA-seq were integrated to identify two CTS QTLs *qCTS8* and *qCTS12*. Furthermore, qRT-PCR, genotype sequencing and knockout analysis indicated that *LOC_Os12g18729* could be the candidate gene of *qCTS12*. These results are expected to further exploration of the genetic mechanism of CTS in rice and improve cold tolerance of cultivated rice by introducing the cold tolerant genes from DXWR through marker-assisted selection.

**Supplementary Information:**

The online version contains supplementary material available at 10.1186/s12870-024-05369-x.

## Background

Rice (*Oryza sativa* L.) is one of the most widely grown food crops in the world, providing for the energy needs for more than half of the global population [1]. Therefore, the sustainable and stable production of rice in Asia, and in particular China, directly promotes global food security. However, cold stress is a major factor restricting the growth and productivity of rice in high-latitude and high-altitude regions with tropical and subtropical climate, as well as in cold, waterlogged paddy fields. Cold damage can occur at any developmental stage in rice, and the seedling stage shows higher sensitivity to cold stress than other developmental stages [2, 3]. In southern China and Yangtze River basin with double rice cropping, cold temperatures in late spring coincide with the early rice growing season, which can cause increased levels of reactive oxygen species and malondialdehyde, damage to cell membranes, changes in proline content, damage to plant photosynthesis, yellowing and eventually necrosis at the early developmental stage [4–7], thus negatively impacting rice plant growth and grain yield. In China, cold stress results in annual losses of 3–5 billion kg in the rice crop [8]. Therefore, screening the cold tolerance of rice germplasm resources and exploring the associated genes are of great practical significance for breeding cold-tolerant rice varieties and stabilizing rice production.

Cold tolerance is a complex quantitative trait controlled by multiple genes. In the past few decades, approximately 270 cold tolerance-related QTLs have been identified in the plants of biparental populations at various growth stages, including over 80 QTLs for CTS [9]. However, a few CTS genes, including *basic region/leucine zipper 73*, *CHILLING TOLERANCE DIVERGENCE 1*, *CHILLING TOLERANCE DIVERGENCE 11*, *Chilling-tolerance in Gengdao/japonica rice 1*, *COLD-TOLERANCE IN GENG RICE 2*, *qCTS-9*, *HAN1* (han termed “chilling” in Chinese), *qPSR10* (PSR: plant survival rate), *WRKY transcription factor 115 *and *lipid transfer protein 159*, were isolated based on QTL mapping and functionally characterized by map based cloning [10–19]. High-resolution single nucleotide polymorphisms (SNPs) genetic map, is one of the most common methods to identify the QTLs and genes related to cold tolerance in rice. For instance, SNP genetic map analyses of a population of 140 BRILs derived from a cross between Dongxiang wild rice (DXWR) and a super rice (SN265) led to the identification of 10 candidate genes related to cold tolerance [20].

Notwithstanding, phenotyping and genotyping numerous individuals from a biparental population entails a major investment in time [9]. In recent years, the development of high-throughput sequencing technologies has enabled efficient, simple and low-cost whole-genome sequencing. BSA was applied to separately pooled DNA of individuals selected from a segregating population, based on their extreme phenotypes, for a target trait using a next-generation sequencing technology. By analyzing the differences in SNPs and insertion/deletion mutations (InDels) between the two groups, it was possible to quickly identify regions closely related to the target trait, making the research results more accurate and reliable [21]. Therefore, BSA-seq is widely used in identifying QTLs for important traits, such as stress tolerance, in rice. For instance, a 96.6-kb region (*qCTS6*) containing 13 annotated genes for CTS was mapped onto chromosome 6 through BSA-seq and next-generation sequencing analyses of an F_2:3_ population derived from a cross between Dongnong430 (chilling sensitive) and Dongfu104 (chilling tolerant). One of the genes in this region, *OsbZIP54*, participated in controlling cold tolerance in rice at the seedling stage [22]. Additionally, BSA-seq analysis of the F_2_ population derived from the cross between the rice cultivars Longjing25 (cold tolerant) and Longjing11 (cold sensitive) led to the identification of a 0.82-Mb region on chromosomes 6 and 9, which was related to cold tolerance at the booting stage. Transcriptome analysis showed that *beta-glucosidase 31* located in this region might be involved in the regulation of cold tolerance at the booting stage [23].

RNA-seq is a measure of mRNA levels in tissues under certain conditions. Evaluation of the types and expression levels of genes in cold stress-treated biological samples under study enables the identification of cold resistance related genes in rice in an efficient manner. RNA-seq analysis of wild rice revealed that genes related to response stimulus, catalytic activity, and uridine diphosphate (UDP) glucosyltransferase were associated with cold tolerance regulation. Overexpression of the UDP glucosyltransferase gene *Low Temperature Growth 5* improved cold tolerance in rice [4]. Comparative transcriptome analyses of DEGs in susceptible and higher in tolerant genotypes showed that signal transformation, photosynthesis, energy and carbohydrate metabolism related genes as well as WRKY, bZIP, NAC, MYB, and other transcription factor family members may be related to cold tolerance in rice [24]. Transcriptome analysis of rice cultivars Oro (cold tolerant) and Tio Taka (cold sensitive) showed that genes involved in phytohormone signal transduction and antioxidant system play an important role in the cold stress response [25]. RNA-seq analysis of the cold-tolerant rice variety P427 revealed that the expression levels of *MYB*, *APETALA2/ethylene-responsive element binding protein*, *NAC*, and *WRKY* family genes as well as genes involved in the ICE (inducer of CBF expression)–CBF (C-repeat binding factor)–COR (cold regulated) pathway were affected by cold stress [26].

In summary, with the development and application of relevant techniques, the molecular mechanisms underlying cold tolerance regulation have been identified in rice. However, these molecular mechanisms were determined mainly in cultivated rice, while those in wild rice remain largely unknown. DXWR grows in the northernmost regions of the world and is an ancestor of Asian cultivated rice. DXWR exhibits greater cold resistance than cultivated rice, and can successfully survive in winter temperatures ranging from 0 °C to as low as-12.8 °C [27]. This suggests that DXWR is a valuable genetic resource for developing cold-tolerant rice cultivars. However, the molecular mechanism of cold tolerance in DXWR remains uncertain, which limits its utilization for developing cold-tolerant rice cultivars. In the present study, three strategies (genetic linkage, BSA-seq and RNA-seq) were employed to identify the cold tolerance-related genes in rice at the early seedling stage. The chilling-tolerant line 19H19 was developed by crossing DXWR with XB. A BC_5_F_2_ population derived from a cross between XB and 19H19 was used for BSA-seq analysis and genetic linkage map construction. The objective of these studies was to investigate the possible association of nucleotide polymorphisms in some candidate genes with the cold tolerance phenotype of rice. Then, RNA-seq was performed to elucidate the transcript levels of candidate genes in XB and 19H19 under normal temperature and severe cold stress, and to identify cold tolerance-associated DEGs. Ultimately, using these approaches, we aimed to infer the potential mechanisms underlying cold stress tolerance in DXWR.

## Results

### Phenotypic evaluation of 19H19, XB and their BC_5_F_2 _progeny under cold stress

**Fig. 1:**
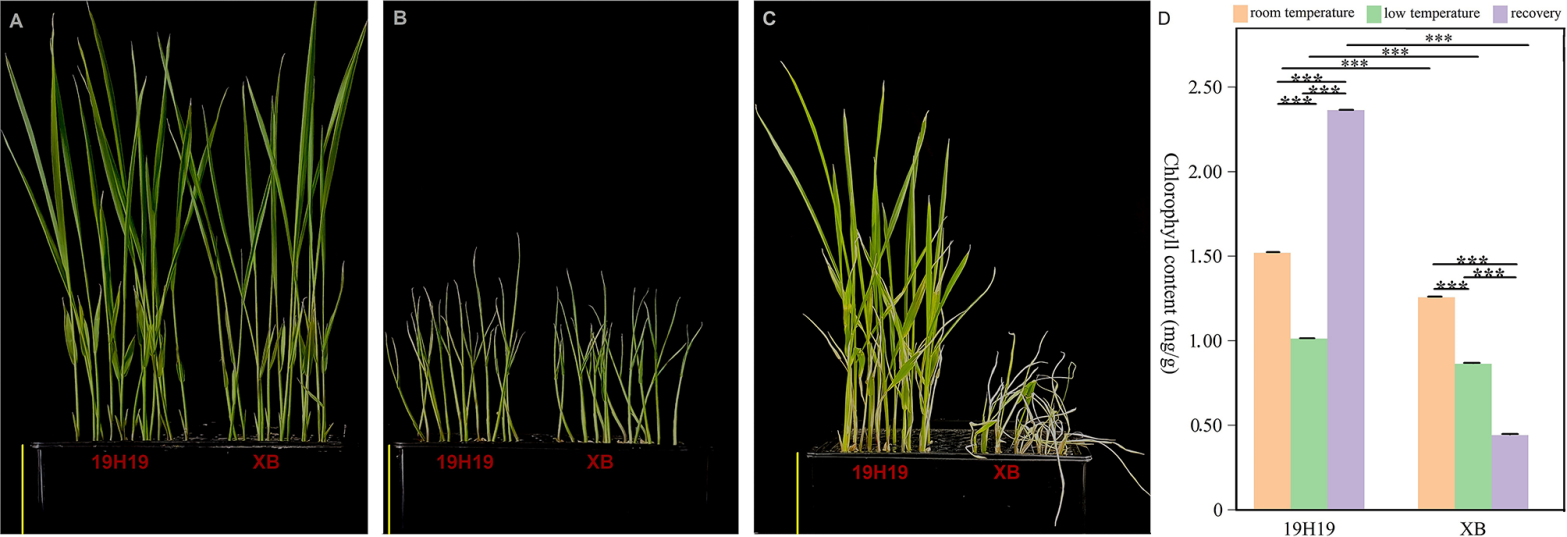
Phenotypes and chlorophyll content of the samples of 19H19 and XB under room/low temperature and recovery treatment. (**A**) The 19H19 and XB seedlings after 4-day room temperature; (**B**) The 19H19 and XB seedlings after 4-day cold treatment; (**C**) The 19H19 and XB seedlings after 7-day recovery treatment; (**D**) Chlorophyll contents of 19H19 and XB under room/low temperature and recovery condition. Scale bars = 5 cm in (**A**), (**B**), and (**C**); Significant differences among different samples were determined using Student’s t-test (***P < 0.001)

After 4 d of RT treatment, no significant differences were observed between the 19H19 and XB plants (Fig. 1A). Similarly, after 4 d of LT treatment, the degree of leaf curling in the 19H19 and XB plants showed no significant variation (Fig. 1A, B). By the end of recovery, the XB seedlings were completely wilted, whereas the 19H19 seedlings showed 96% of survival rate (Fig. 1C).In addition, the chlorophyll content of 19H19 seedlings was significantly higher than that of XB seedlings, regardless of the temperature (normal or low) and subsequent recovery (Fig. 1D). The chlorophyll content of XB seedlings decreased continuously during the period of LT treatment and subsequent recovery, whereas that of 19H19 seedlings increased after the 7-day recovery treatment (Fig. 1D). Photosynthetic activity in rice plants was greatly diminished when subjected to cold. Generally, a strong correlation is observed between chlorophyll content and photosynthetic activity.

**Fig. 2:**
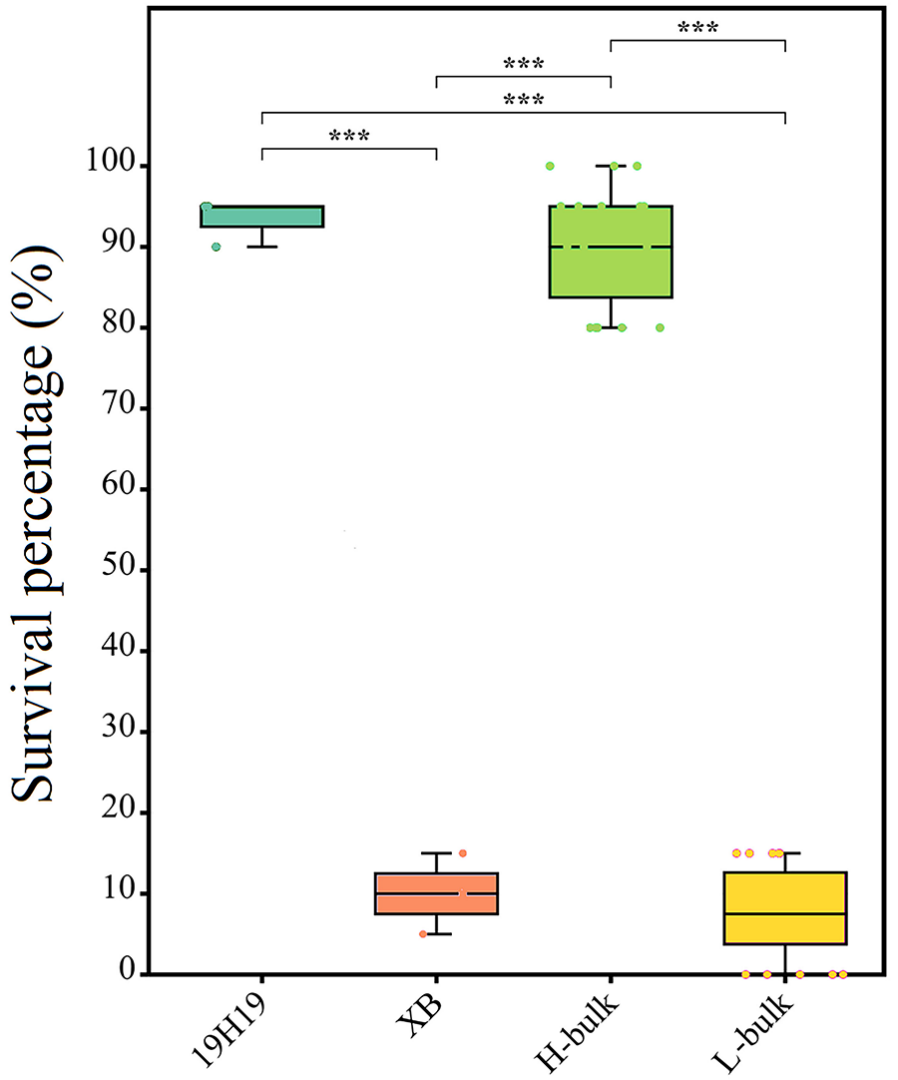
Boxplots represent SP of H-bulk, L-bulk and parental lines. The band inside the boxes indicates the median. Significant differences among diferent samples were determined using Student’s t-test (***P < 0.001)

The seedling SP values of 120 BC_5_F_2_ lines and their parents (19H19 and XB) ranged from 0 to 100%, with an average SP value of 47%. Twenty BC_5_F_2_ lines with SP values higher than 80% and consistently showing high cold tolerance in three LT treatments were pooled as H-bulks, and another 20 backcross inbred lines (BILs) with SP values lower than 15% and consistently showing low cold tolerance were pooled as L-bulks (Fig. 2).

### Differences in the transcriptomes of 19H19 and XB seedlings during the cold treatment and subsequent recovery

Transcriptome is a collection of all RNA molecules in a cell or group of cells, which reflects the expression status of the entire genome. The transcriptome data and quality of 19H19 and XB seedlings during the period of RT/LT treatment and subsequent recovery are shown in Table S1. A total of 58,666 genes were identified in these datasets (Table S2). Of these, approximately 38,000 genes showed FPKM values ranging from 0 to 1; approximately 8,000 genes showed FPKM values ranging from 3 to 15; and between 1,243 and 1,669 genes showed FPKM values over 60 (Table S2).

**Fig. 3:**
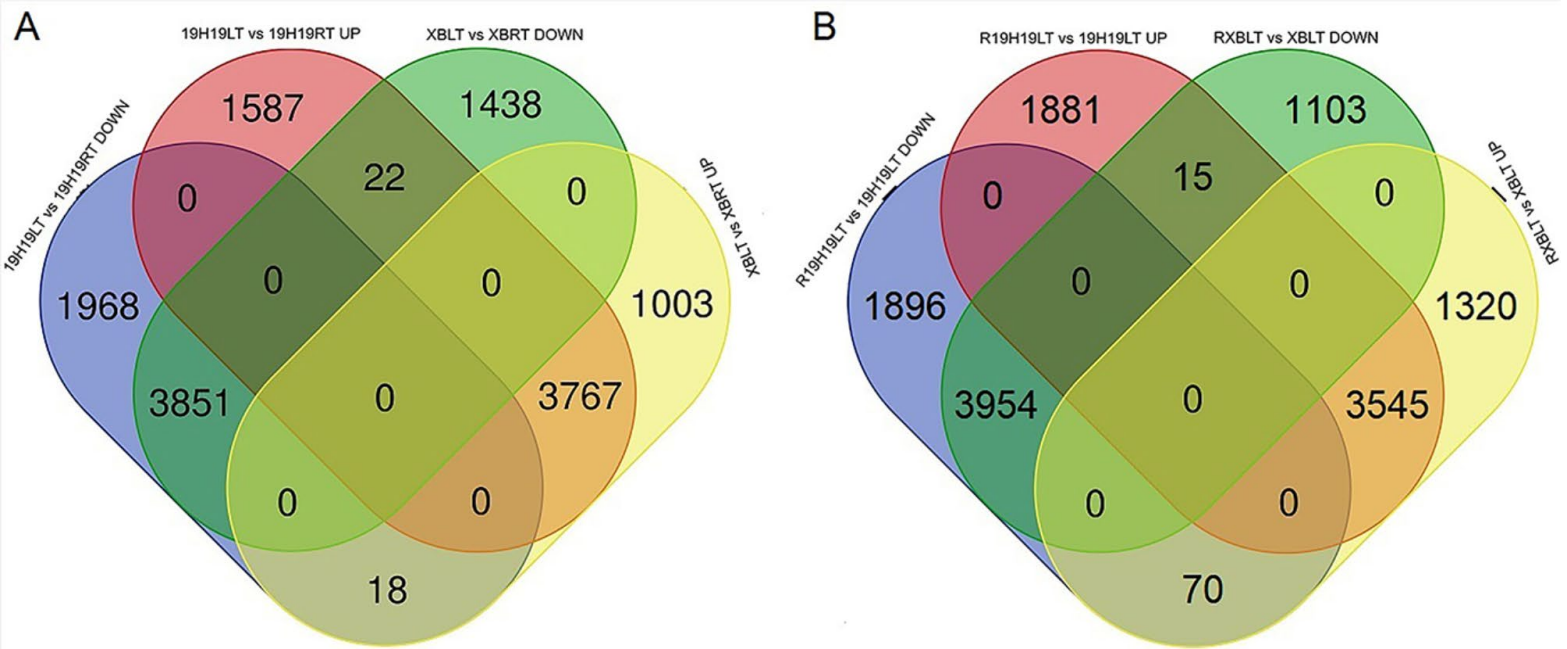
Venn diagram showing the numbers of DEGs identified in 19H19 and XB in the room/low temperature treatment or during the subsequent recovery period. (**A**) Numbers of DEGs identified in the LT vs RT treatments of 19H19 seedlings (19H19LTvs19H19RT) and XB seedlings (XBLTvsXBRT). (**B**) Numbers of DEGs identified in the LT vs recovery (R) treatments of 19H19 seedlings (R19H19LTvs19H19LT) and XB seedlings (RXBLTvsXBLT)

The number of down- and up-regulated genes differed between the 19H19 and XB seedlings, regardless of the temperature and subsequent recovery. Numbers of down-regulated genes identified between the LT and RT treatments in 19H19 seedlings (19H19LTvs19H19RT) and XB seedlings (XBLTvsXBRT) were 5,837 and 5,311, respectively, of which 3,851 were common to both groups (Fig. 3A). Numbers of up-regulated genes identified in the 19H19LTvs19H19RT and XBLTvsXBRT comparisons were 5,376 and 4,788, respectively, of which 3,767 were common to both groups (Fig. 3A).

**Fig. 4:**
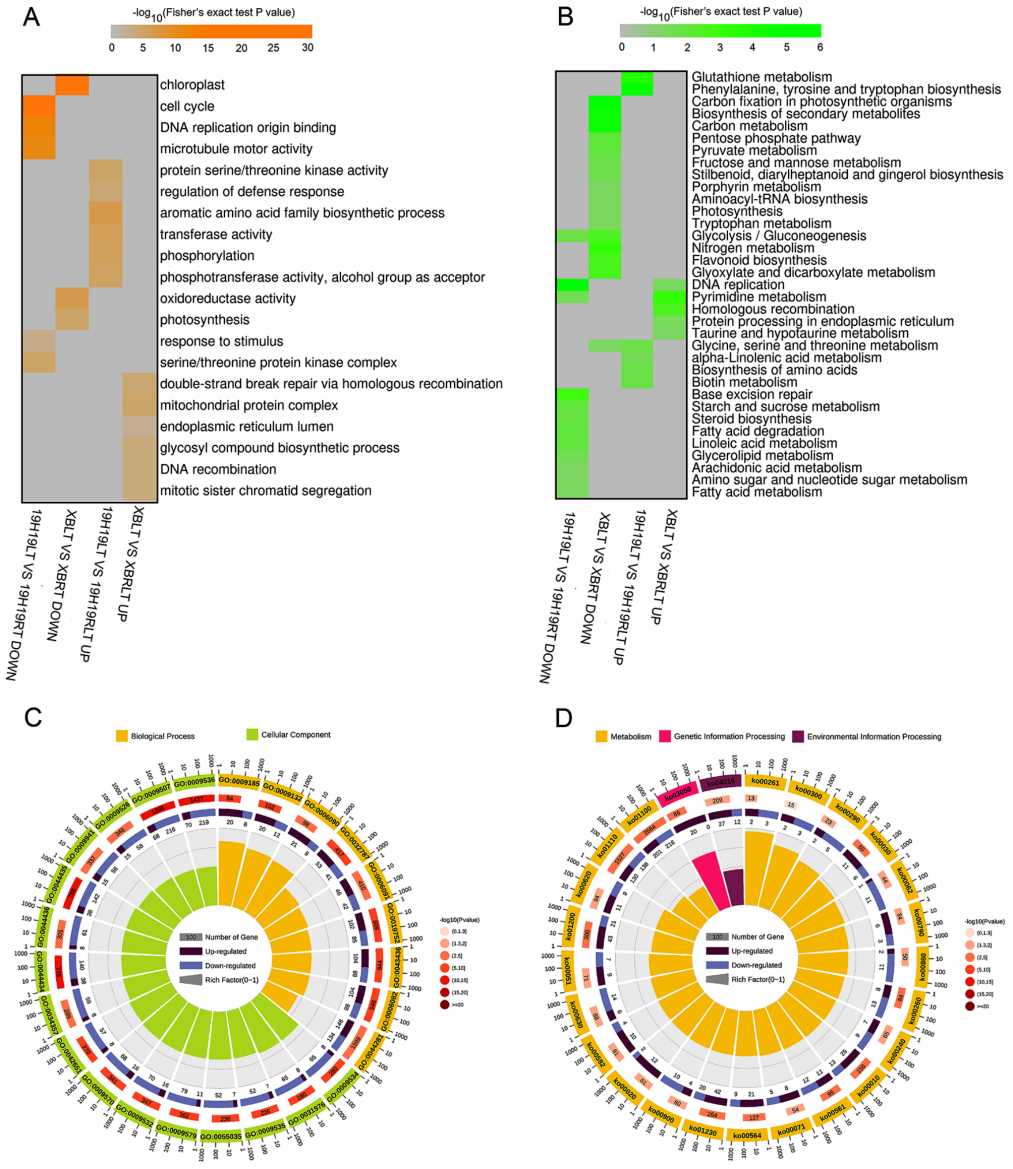
GO and KEGG enrichment analyses of DEGs identified in the 19H19LTvs19H19RT and XBLTvsXBRT groups. (A, B) GO (**A**) and KEGG (**B**) enrichments of DEGs unique to the 19H19LTvs19H19RT and XBLTvsXBRT groups. (C, D) GO (**C**) and KEGG (**D**) enrichments of DEGs common to the 19H19LTvs19H19RT and XBLTvsXBRT groups

Based on the annotated genes in rice, the 19H19LTvs19H19RT and XBLTvsXBRT showed different GO and KEGG enrichments for the unique down- and up-regulated genes (Fig. 4A, B). The results of GO enrichment analysis of the DEGs are shown in Fig. 4A. In each GO category, the terms enriched among the DEGs were significantly different between the 19H19LTvs19H19RT and XBLTvsXBRT groups. For example, ‘phosphorylation’ and ‘phosphotransferase activity, alcohol group as acceptor’ were mainly enriched among some upregulated genes unique to the 19H19LTvs19H19RT group. Studies show that phosphoproteins play an important role in mediating cold stress acclimation in rice [28]. In this study, the 19H19 seedlings exhibited greater cold resistance than XB seedlings. This suggests that genes involved in the phosphorylation related pathway play key roles in the regulation of cold tolerance in rice. The results of KEGG pathway enrichment analysis showed that ‘DNA replication’ (ko03030) and ‘pyrimidine metabolism’ (ko00240) pathways were collectively enriched among some down-regulated genes unique to the 19H19LTvs19H19RT group and upregulated genes unique to the XBLTvsXBRT group, while the ‘glycine, serine and threonine metabolism’ pathway was enriched among some up-regulated genes unique to the 19H19LTvs19H19RT group and down-regulated genes unique to the XBLTvsXBRT group (Fig. 4B). In addition, the DEGs common to 19H19LTvs19H19RT and XBLTvsXBRT groups were mainly enriched in 30 GO terms, including ‘pyruvate metabolic process’ (GO:0006090), ‘chloroplast thylakoid membrane’ (GO:0009535) and ‘photosynthetic membrane’ (GO:0034357), among others (Fig. 4C). Interestingly, the physiological process of photosynthesis is sensitive to environmental temperature [29]. The downregulated genes common to both the 19H19LTvs19H19RT and XBLTvsXBRT groups and those unique to the XBLTvsXBRT group were mainly enriched in the chloroplastic-related pathway (Fig. 4A, C), implying that the expression of chloroplast genes is downregulated under the influence of cold stress, while the down-regulation of more chloroplastic-related genes in XB, which might result the material to be sensitive to cold stress. KEGG enrichment analysis showed that the DEGs identified above were significantly enriched (*P* < 0.05) in 25 pathways, namely, ‘Citrate cycle (TCA cycle)’ (ko00020), ‘Fatty acid elongation’ (ko00062), ‘Porphyrin metabolism’ (ko00860) and ‘Proteasome’ (ko03050) (Fig. 4D). Overall, GO and KEGG enrichments of DEGs were different between 19H19 and XB seedlings, which ultimately led to differences in their cold resistance capacity.

Moreover, the number of down- and up-regulated genes differed between 19H19 and XB, regardless of the period of recovery. A total of 5,920 and 5,072 genes were downregulated in the LT treatment vs. the recovery (R) treatment in 19H19 seedlings (R19H19LTvs19H19LT) and XB seedlings (RXBLTvsXBLT), respectively, of which 3,954 overlapped between the two groups (Fig. 3B). Similarly, a total of 5,441 and 4,935 up-regulated genes were identified in R19H19LTvs19H19LT and RXBLTvsXBLT, respectively, of which 3,545 overlapped between the two groups (Fig. 3B).

**Fig. 5:**
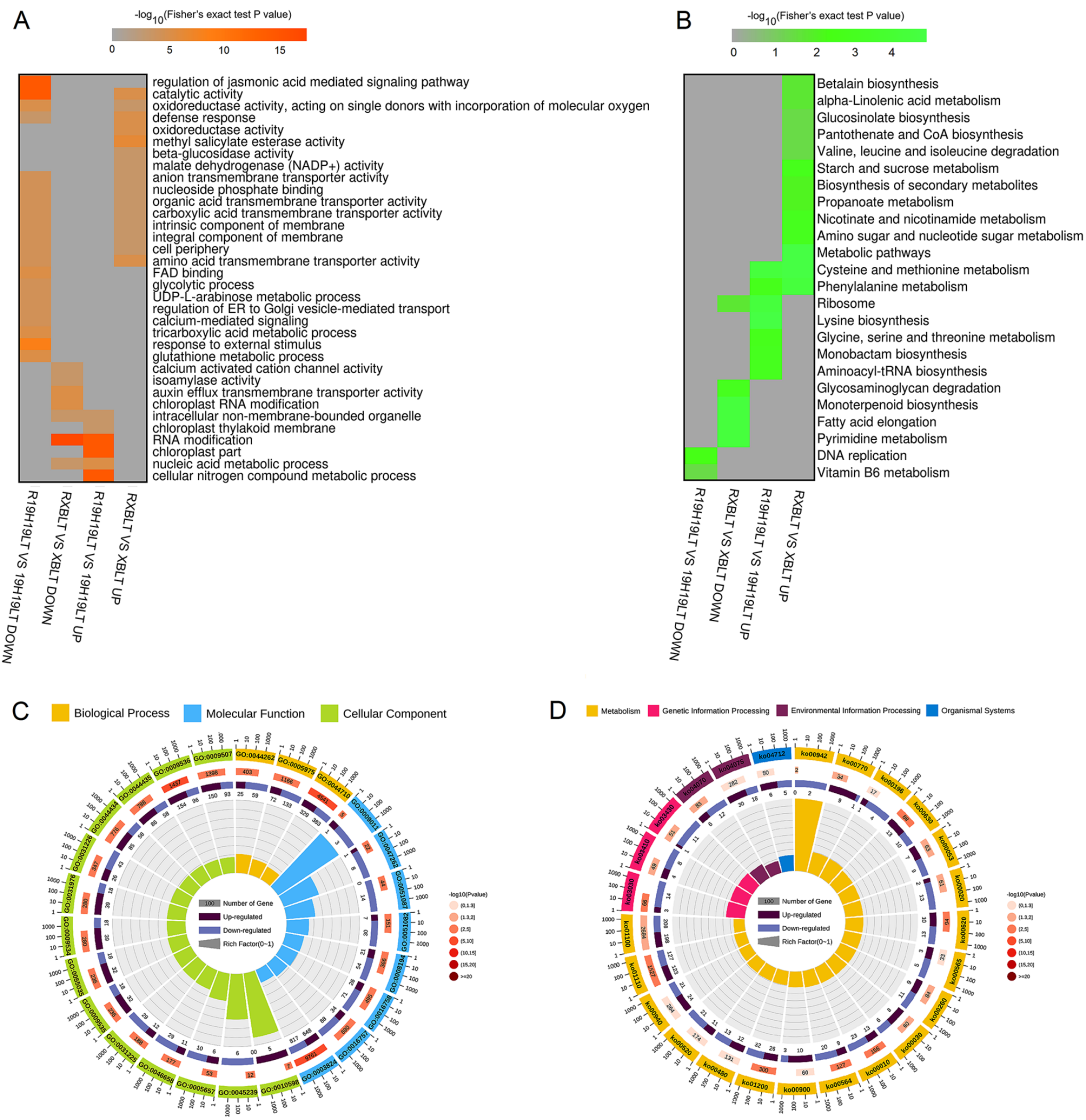
GO and KEGG enrichment analyses of DEGs identified in the R19H19LT vs. 19H19LT and RXBLT vs. XBLT groups. (A, B) GO (**A**) and KEGG (**B**) enrichments of DEGs unique to the R19H19LT vs 19H19LT and RXBLT vs XBLT groups. (C, D) GO (**C**) and KEGG (**D**) enrichments of DEGs common to both R19H19LT vs 19H19LT and RXBLT vs XBLT groups

Eleven GO terms, such as ‘anion transmembrane transporter activity’, ‘nucleoside phosphate binding’ and‘defense response’, were collectively enriched among some down-regulated genes unique to the R19H19LTvs19H19LT group or upregulated genes unique to the RXBLTvsXBLT group. Similarly, ‘intracellular non-membrane-bounded organelle’, ‘RNA modification’, and ‘nucleic acid metabolic process’ were collectively enriched among some upregulated genes unique to the R19H19LTvs19H19LT group or down-regulated genes unique to the RXBLTvsXBLT group (Fig. 5A). Only the ‘ribosome’ pathway was collectively enriched among some upregulated genes unique to the R19H19LTvs19H19LT group or downregulated genes unique to the RXBLTvsXBLT group (Fig. 5B). The DEGs common to R19H19LTvs19H19LT and RXBLTvsXBLT groups were mainly enriched in 30 GO terms, including ‘NAD(P)H dehydrogenase complex (plastoquinone)’ (GO:0010598) and ‘chaperone binding’, and GO:0045239 ‘tricarboxylic acid cycle enzyme complex’(GO:0051087), among others (Fig. 5C). The downregulated genes common to R19H19LTvs19H19LT and RXBLTvsXBLT groups were mainly enriched in ‘tricarboxylic acid cycle enzyme complex’ and ‘chaperone binding’, and the upregulated genes common to R19H19LTvs19H19LT and RXBLTvsXBLT groups were mainly enriched in ‘NAD(P)H dehydrogenase complex (plastoquinone)’. KEGG enrichment analysis showed that the DEGs identified above were significantly enriched (*P* < 0.05) in 17 pathways. Among these pathways, the ‘Citrate cycle (TCA cycle)’ (ko00020), ‘anthocyanin biosynthesis’ (ko00942), ‘mismatch repair’ (ko03430), ‘Base excision repair’ (ko03410), ‘DNA replication’ (ko03030), ‘Glycerophospholipid metabolism’ (ko00564), and ‘Glycolysis/Gluconeogenesis’ (ko00010) pathways were enriched among the downregulated genes common to both R19H19LTvs19H19LT and RXBLTvsXBLT groups, while ‘Pantothenate and CoA biosynthesis’ (ko00770), and ‘Plant hormone signal transduction’ (ko04075) pathways were enriched among the up-regulated genes common to both R19H19LTvs19H19LT and RXBLTvsXBLT groups (Fig. 5D).

### Comprehensive QTL mapping for cold tolerance in BILs population

**Fig. 6:**
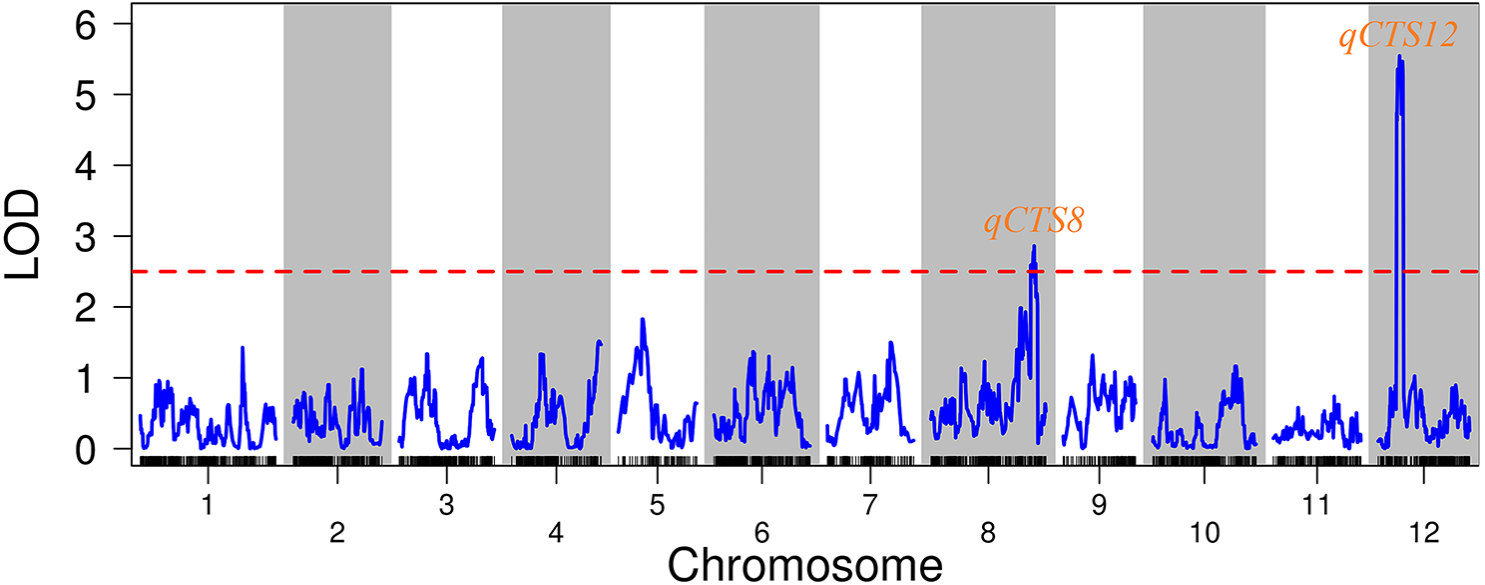
QTL mapping based on seedling survival percentage (SP)

Based on genotyping using high-density bin markers, the 19H19 genotype is 82.9% identical to XB, 3.5% identical to DXWR, and 13.6% is heterozygous. It was indicated that 19H19 show high genetic homogeneity with their recurrent parent XB (Fig. S1). These consistent bins were filtered out. The 2,059 SNP markers were used in QTL mapping of cold tolerance based on SP. The total genetic distance of linkage map was 1645.884 cM, with the individual linkage groups ranging from 104.541 to 196.041 cM in length (Table S3). An LOD threshold of 2.5 was used to identify QTLs for the trait. Two QTLs for SP were detected on chromosomes 8 and 12, respectively (Fig. 6). Among them, *qCTS12* in the physical interval of 10,786,555–11,988,730 bp on chromosome 12 had LOD score of 5.55 and explained 23.9% of total phenotypic variance (Table 1). To further validate the *qCTS12*, three recombinant plants with sequential residual heterozygous (RH) segments in the interval of RM3246-RM27956, were selected from the BC_5_F_2_ generation. They were selfed to produce three corresponding BC_5_F_3_ RH populations, respectively (Fig. S2). Results of QTL detected for CTS using three RH populations are shown in Table S4. Significant QTL effects were detected in B1, B2 and B3. The additive effects were 20.25%, 19.25% and 14.04%, explaining 22.80%, 41.78% and 22.67% of the phenotypic variance, respectively. The enhancing alleles of SP were derived from DXWR in both populations. These results showed that *qCTS12* was located within a 702.5 kb region flanked by markers RM27940 and Tw10811, which segregating region both in B1, B2 and B3 populations.

**Table 1:** QTL summary under cold stress for SP

QTL	Chr	LOD	PVE	A	Marker interval
*qCTS12*	12	5.55	23.9	17.63	12.10786555–12.11988730
*qCTS8*	8	2.86	10.67	12.03	8.27223710–8.28008078

A, additive effect of replacing a XB allele with an DXWR allele; PVE, phenotypic variance explained

### BSA-Seq data analysis and reads assembly

Approximately 3,548 Mbp raw read data were acquired by the BSA-seq of four sequencing libraries using the Illumina TruSeq platform. The raw reads were filtered, and approximately 3,539 Mbp clean reads (99.74%) were obtained. The effective rates were 99.69%, 99.77%, 99.73%, and 99.74% in XB, L-bulks, 19H19 and H-bulks, respectively. Moreover, the sequencing depths were 9.14-, 29.09-, 10.09- and 27.11-fold in XB, L-bulks, 19H19 and H-bulks, respectively (Table 2). This indicated that the sequencing depth was higher in segregating pools than in parents (Table 2).

**Table 2:** Overview of the BSA-Seq data

Sample	Raw Base(bp)	Clean Base(bp)	Effective Rate(%)	Error Rate(%)	Average depth(X)	Coverage at least 1X(%)
XB	4,074,712,800	4,062,164,400	99.69	0.03	9.14	89.51
L-bulks	13,990,471,200	13,957,730,400	99.77	0.03	29.09	94.41
19H19	4,653,923,100	4,641,387,600	99.73	0.03	10.09	90.10
H-bulks	12,763,384,800	12,730,228,500	99.74	0.03	27.11	95.36

### Analysis of expression profiles and identification of candidate regions related to cold tolerance at the early seedling stage

**Fig. 7:**
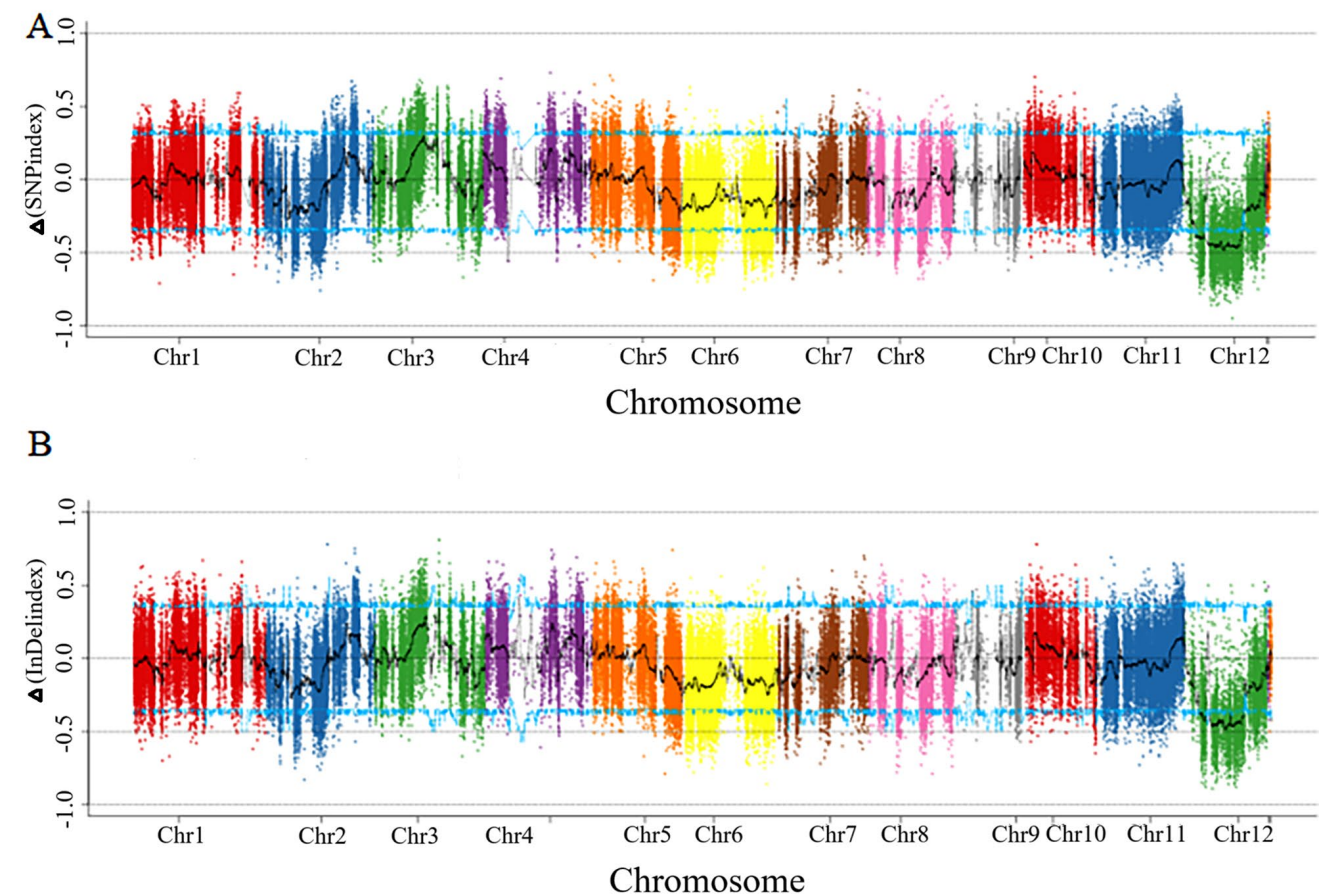
Manhattan plot analysis on the variation tendency of the ΔSNP index and Δindel index between H-bulk and L-bulk associated with SP distribution in the chromosomes: (**A**) ΔSNP index and (**B**) Δindel index. The number of X-axis represents the chromosome number. The blue curve lines represent the threshold of ΔSNP index and Δindel index

**Fig. 8:**
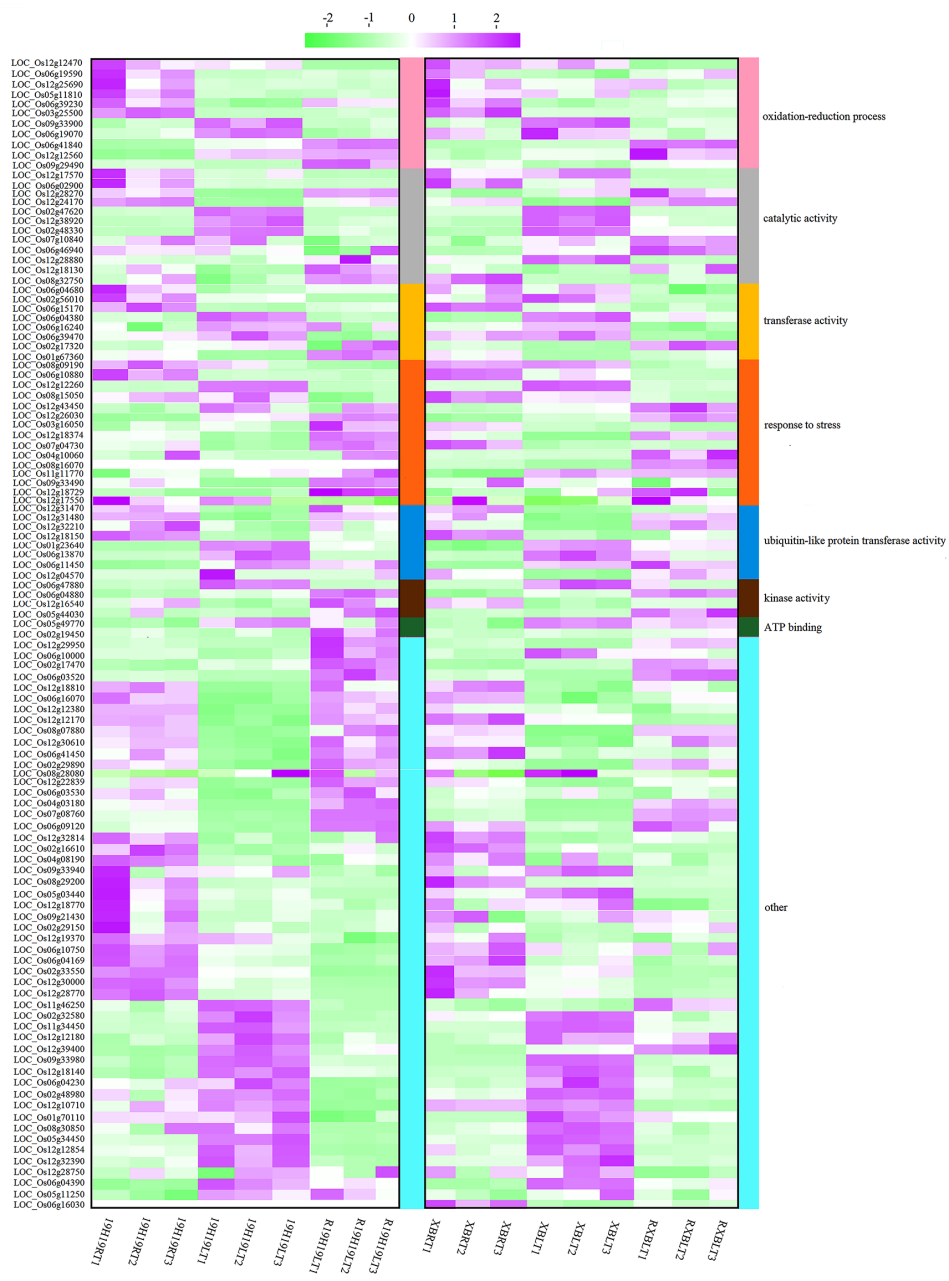
Heat map showing the expression levels of candidate genes in 19H19RT, 19H19LT, R19H19LT, XBRT, XBLT, and RXBLT leaves

Based on the results of BSA-seq, candidate regions in the rice genome related to cold tolerance at the early seedling stage were detected using the association analysis of ΔSNP index and Δindel index. The candidate region for BSA-seq is located at 9,915,001–18,852,000 bp on chromosome 12. Due to the large size of the interval, the region (*qCTS12*) simultaneously detected by both GBS-seq and BSA-seq was selected for validation. To avoid ignoring the impact of minor QTLs, the selection of candidate genes (SNPs and Indels) was performed throughout the genome. SNPs causing non-synonymous substitutions in coding regions or indels in promoter regions of these genes were found (Table S5), which may have phenotypic effects. A total of 112 candidate genes were selected, most of which were located on chromosome 12 (Figs. 7 and 8). The interval located by BSA-seq coincided with the interval of *qCTS12* localized under cold stress (Figs. 6 and 7). GO enrichment analysis indicated that 60 out of 112 genes were related to functional categories such as ‘oxidation-reduction process’, ‘transferase activity’, ‘catalytic activity’, ‘response to stress’, ‘ubiquitin-like protein transferase activity’, ‘kinase activity’, and ‘ATP binding’. Changes in the expression trends of most of these candidate genes in the RT /LT treatment and during recovery period were identical in both 19H19 and XB. Only some candidate genes related to ‘oxidation-reduction process’ were downregulated in 19H19 but upregulated in XB after cold stress. Some candidate genes related to ‘response to stress’ showed no significant change in the expression in 19H19 after cold stress, but their expression was up-regulated in XB. Furthermore, we have listed the top 20 upregulated and top 20 downregulated genes in (19H19LTvs19H19RT and XBLTvsXBRT) / (R19H19LTvs19H19LT and RXBLTvsXBLT). Among these genes, *LOC_Os08g43700* falls within the *qCTS8* interval. In addition, we found that out of these top regulated genes, 9 are associated with ‘oxidation-reduction processes’, and another 9 are related to ‘responses to stress’ (Table S6). These results suggest that the difference in the expression profiles of these two functional (‘oxidation-reduction process’ and ‘response to stress’) candidate genes may be responsible for the difference in the cold tolerance of these two genotypes (Fig. 8).

### Interactions/coexpression of candidate genes with other functional genes regulates cold tolerance in rice

**Fig. 9:**
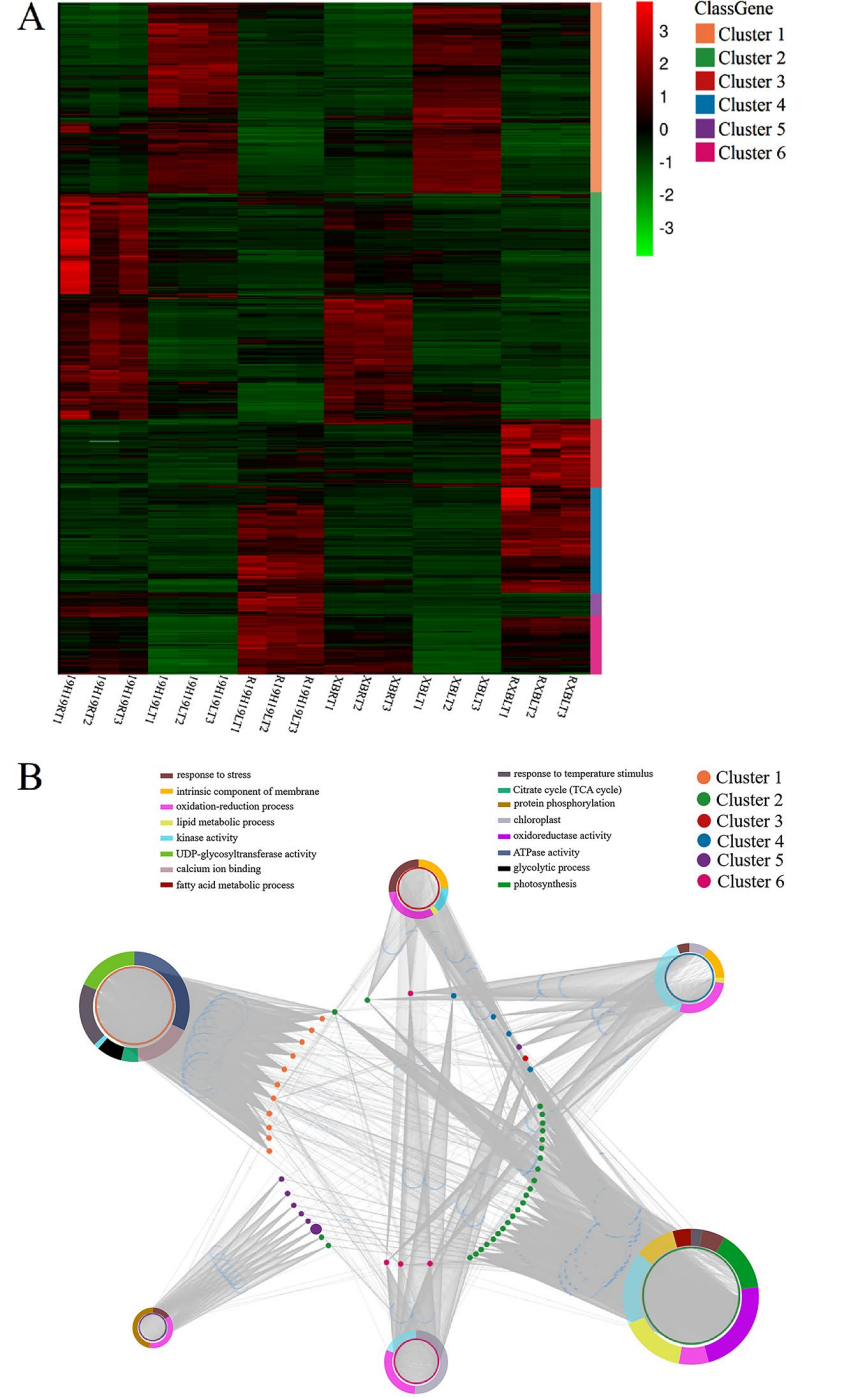
Hierarchical clustering of quantified genes and the interactions between candidate genes (identified by BSA-seq) and DEGs (identified by RNA-seq) in room-, cold- and recovery-treated 19H19 and XB seedlings. (**A**) Hierarchical clustering of quantified genes based on three replicates of RNA-seq data. (**B**) Gene interaction network involved in cold tolerance regulation in rice. Nodes in the middle represent candidate BSA-seq genes. Colors of different boxes represent the major functional categories of genes. Colors of different nodes represent the different clusters

To further explore the molecular mechanism of cold tolerance in rice, the interactions/coexpression of candidate genes with other functional genes were analyzed using the MERLIN algorithm. The MERLIN algorithm is based mainly on a large transcriptome data set and is used to predict the regulation of gene expression by the interactions between candidate genes (target genes) and regulatory factors [30]. The candidate genes identified by BSA-seq in this study were considered as target genes, and DEGs identified by RNA-seq were considered as factors. In this study, 112 target genes and 7,289 factors were used as the input for the MERLIN algorithm. The results revealed 806 interactions among 102 factors and 25 target genes (Table S7). Additionally, a coexpression network was constructed using the WGCNA package of R [31] with 7,289 expressed genes, followed by decomposition of the network into 11 sub-network modules (Table S8). Each module contains a set of genes showing significant expression correlations with each other. In the integrated MERLIN and WGCNA network, 53 candidate BSA-seq genes and more than 1,549 DEGs were identified in the leaves of the six rice samples (Fig. 9).

According to the expression patterns of DEGs identified in 19H19 and XB seedlings in the RT/LT treatment or subsequent recovery, these genes are divided into six clusters. Genes belonging to ‘cluster 1’, ‘cluster 2’ and ‘cluster 4’ showed identical expression patterns in 19H19 and XB seedlings (Fig. 9A). In the network, most genes belonged to‘cluster 1’ and ‘cluster 2’. The network contained at least 16 functional categories, such as ‘response to stress’, ‘oxidation-reduction process’, ‘chloroplast’, ‘TCA cycle’, ‘protein phosphorylation’, ‘response to temperature stimulus’, ‘photosynthesis’, and ‘kinase activity’(Fig. 9B). Most of the candidate BSA-seq genes showed significant direct interactions with the functional DEGs in the ‘oxidation-reduction process’, ‘kinase activity’, and ‘chloroplast’ categories (Fig. 9B). It has been reported that genes related to ‘oxidation-reduction process’, ‘kinase activity’, and ‘chloroplast’ are involved in the regulation of cold tolerance in rice [32–34]. These results suggest that candidate genes identified by BSA-seq regulate rice cold tolerance through interaction with functional DEGs.

### Verification of RNA-seq data

**Fig. 10:**
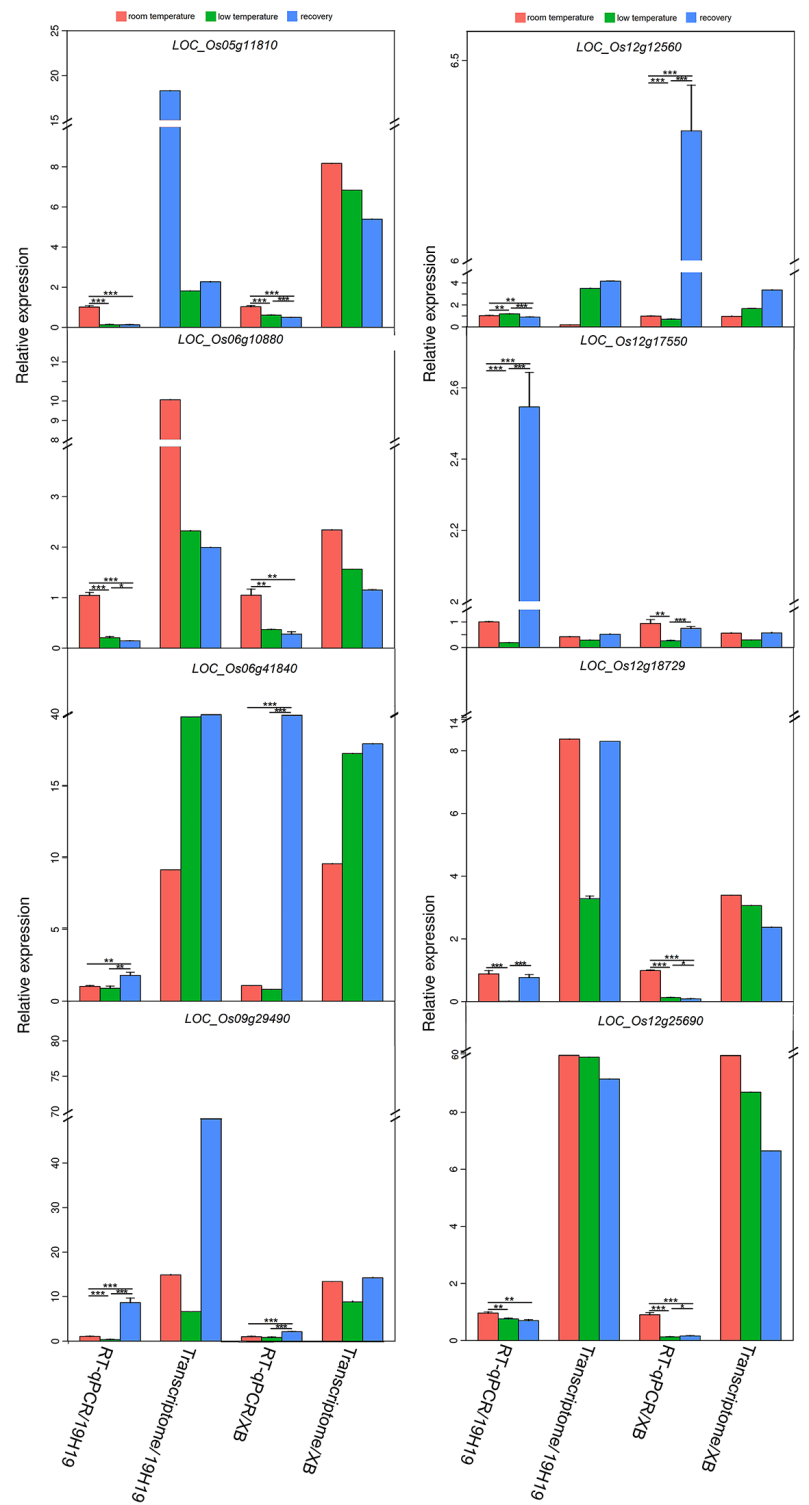
Validation of the RNA-seq data of 8 differentially expressed candidate genes of BSA by RT-qPCR. Data represent mean ± SD (*n* = 3). Significant differences in gene expression levels among the three rice genotypes were determined by Student’s test (*P < 0.05, **P < 0.01, ***P < 0.001). The expression level of each gene in room temperature 19H19 and XB leaves were defined as 1

To verify the reliability of RNA-seq data, the expression of eight candidate DEGs with ‘oxidation-reduction process’ or ‘response to stress’ functions was analyzed by RT-qPCR using the same batch of leaves as those used for RNA-seq. Of these eight genes, five were included in the gene interaction/coexpression network (Figs. 8 and 9B). The RT-qPCR results of six genes (*LOC_Os06g10880*, *LOC_Os12g17550*, *LOC_Os12g25690*, *LOC_Os09g29490*, *LOC_Os12g18729*,and *LOC_Os05g11810*) were consistent with the RNA-seq data, indicating that our transcriptome data are reliable (Fig. 10). The RT-qPCR results of the other two genes were inconsistent with the RNA-seq data, possibly because of differences in the sensitivity of the two methods [35].

Expression levels of the above-mentioned eight genes were significantly affected by the LT treatment (Fig. 10), further implying that these genes are related to the cold tolerance of rice. Expression levels of *LOC_Os06g10880*, *LOC_Os12g25690* and *LOC_Os05g11810 *decreased constantly in 19H19 and XB seedlings during the LT treatment and subsequent recovery, whereas those of *LOC_Os09g29490*, *LOC_Os06g41840*, and *LOC_Os12g12560* continued to increase. These genes showed identical expression patterns in 19H19 and XB. However, the expression patterns of *LOC_Os12g18729* and *LOC_Os12g17550* were different between 19H19 and XB seedlings during the period of recovery; recovery treatment restored and enhanced the expression levels of these genes in 19H19 but weakened their expression levels in XB. In addition, expression level of *LOC_Os12g17550* was the highest in 19H19 after the recovery treatment, while its expression level was the highest in XB in the RT treatment.

### Verification of candidate genes

**Fig. 11:**
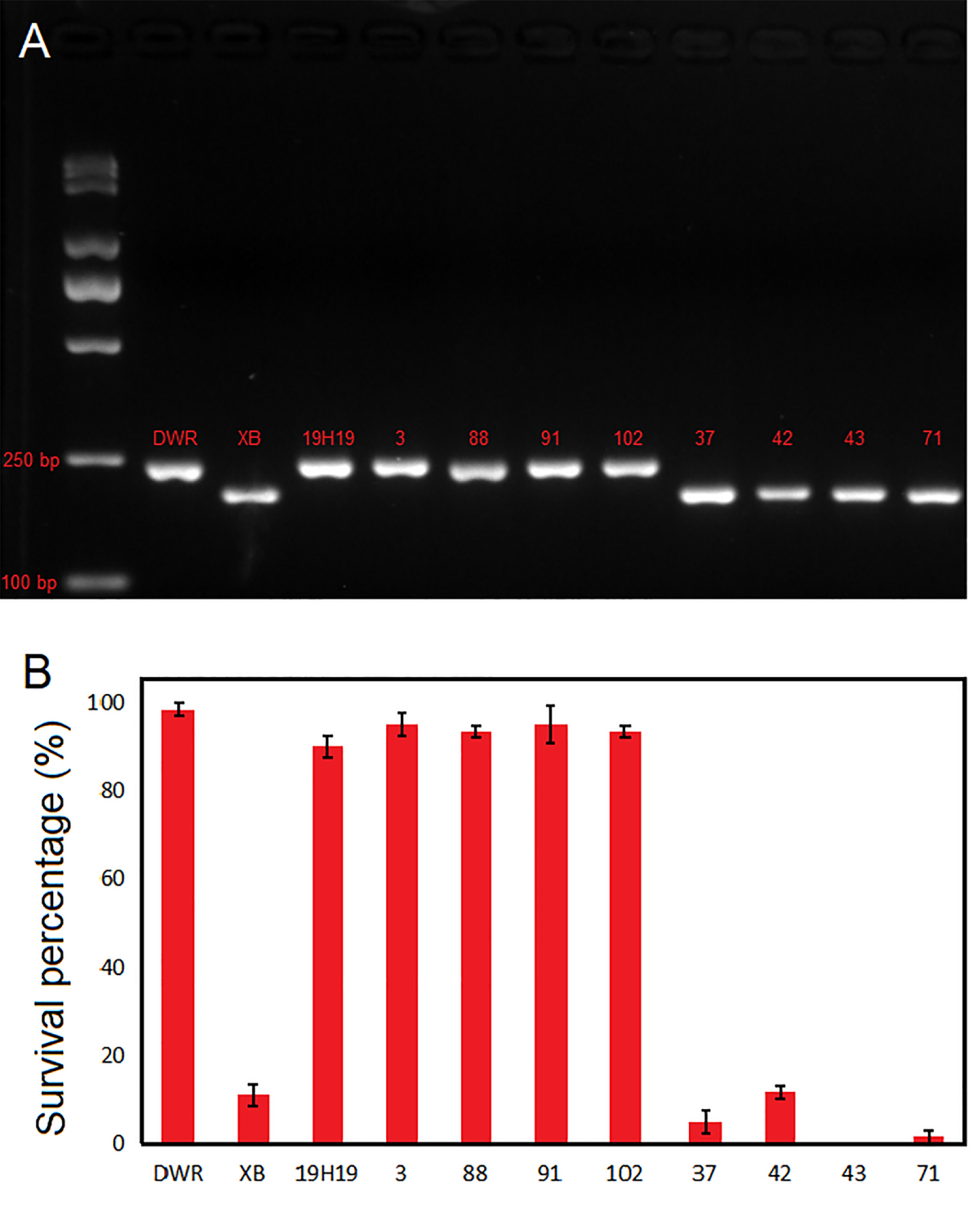
Genotype and SP of DXWR, XB, 19H19, and BC_5_F_2_ individuals. (A, B) Genotype (**A**) and SP values (**B**) of DXWR, XB, 19H19, and BC_5_F_2_ individuals

The expression levels of *LOC_Os12g18729* and *LOC_Os12g17550 *varied between 19H19 and XB seedlings. And the two genes were located in the interval of *qCTS12.* Therefore, we analyzed the sequences of *LOC_Os12g18729* and *LOC_Os12g17550* in 19H19 and XB. Compared with 19H19, the XB seedlings showed a 42-bp deletion in the third exon of *LOC_Os12g18729* and a 7-bp insertion in the third exon of *LOC_Os12g17550* (Fig. S3). Therefore, we analysed the LOD score and PVE for the 42 bp deletion and the 7 bp insertion. Interestingly, the LOD value for the 42 bp deletion is 5.42, with a PVE of 32.47 (Table 3). And the genotypes of 19H19 and BC_5_F_2_ individuals with SP values higher than 85% were consistent with DXWR, while the genotypes of BC_5_F_2_ individuals with SP values lower than 15% were consistent with XB (Fig. 11, Fig. S4). Meanwhile, the LOD value for the 7 bp insertion is 1.91, with a PVE of 25.93 (Table 3). These results further suggest that *LOC_Os12g18729* is a candidate gene for cold tolerance in rice early seedling stage.

**Table 3:** Validation of mapping for markers *LOC_Os12g18729* and *LOC_Os12g17550* in the entire population

Gene ID	Chr.	LOD	*A*	PVE
*LOC_Os12g18729*	12	5.42	23.62	32.47
*LOC_Os12g17550*	12	1.91	16.42	25.93

A, additive effect of replacing a XB allele with an DXWR allele; PVE, phenotypic variance explained

### Verifcation of the function of cold tolerance-related gene *LOC_Os12g18729*

**Fig. 12:**
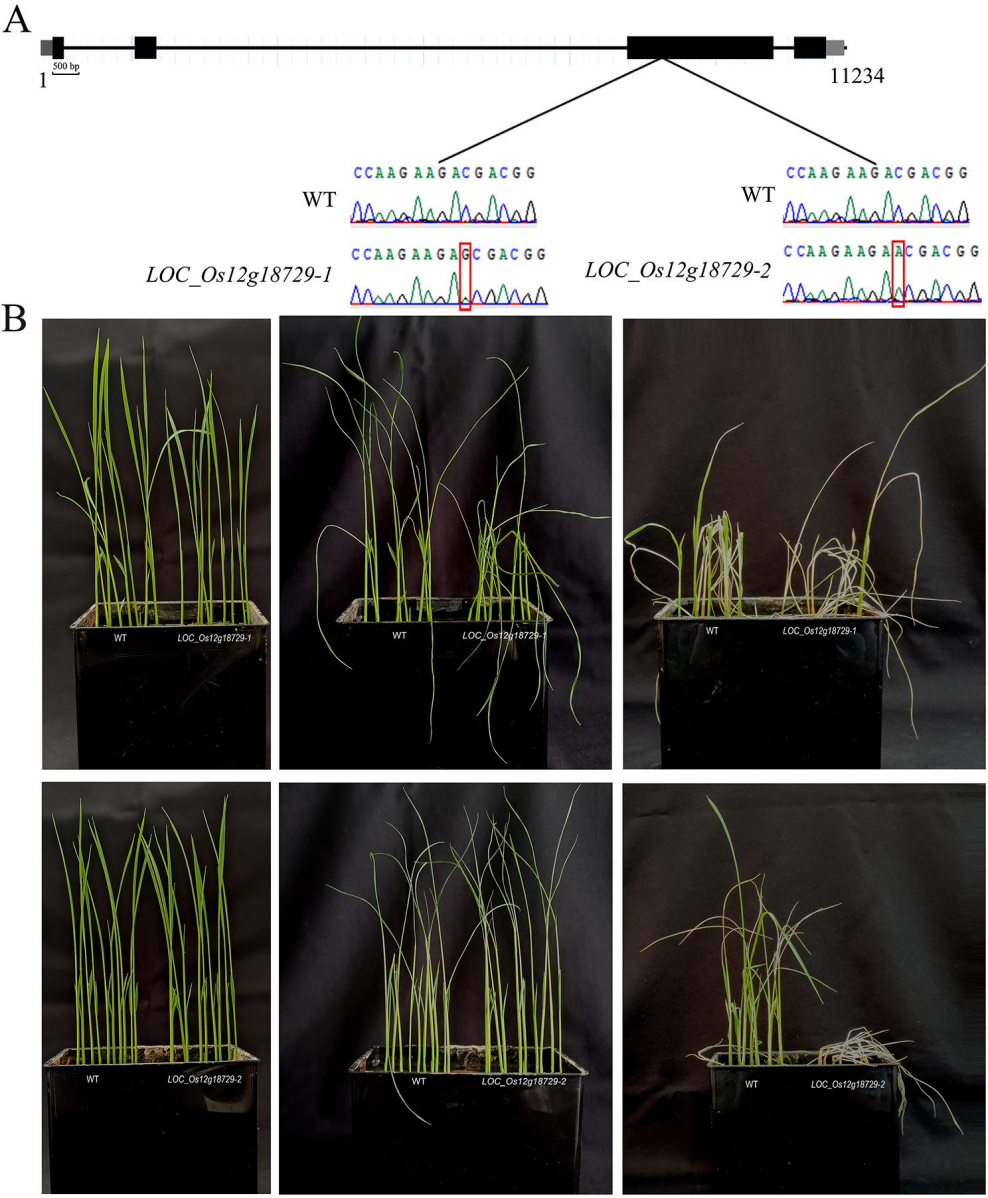
Effect of *LOC_Os12g18729* knockout on the cold tolerance of rice ‘Nipponbare’. (**A**) Target sites in the WT. Inserted nucleotide are marked in red box. (**B**) The WT and mutant seedlings after 7-day cold treatment and 7-day recovery treatment

To verify the function of *LOC_Os12g18729*, a knockout was performed at the site of a 42 bp deletion in *LOC_Os12g18729*. Two knockout mutants (a homozygous line *LOC_Os12g18729*-1 and a homozygous line *LOC_Os12g18729*-2) were generated in the background of ‘Nipponbare’ through the CRISPR/Cas9 system. The *LOC_Os12g18729*-1 contained a “G” insertion, and the *LOC_Os12g18729*-2 contained a “A” insertion (Fig. 12A). Similarly, after 7 d of LT treatment, the degree of leaf curling in the wild type (WT) and mutant plants showed no significant variation (Fig. 12B). By the end of recovery, the mutant seedlings were almost entirely wilted, whereas the WT seedlings showed 65% survival (Fig. 12B), suggesting the disruptions of *LOC_Os12g18729* gene significantly decreased cold tolerance in early rice seedling stage.

## Discussion

Rice is more sensitive to cold stress than other cereal crops, especially when the cold weather in late spring coincides with the early developmental stages of rice seedlings. Moreover, low water and soil temperature in cold waterlogged paddy fields greatly reduce rice yield [36]. Therefore, understanding the molecular mechanisms of rice cold tolerance, exploring rice cold tolerance-related genes and developing varieties with high cold tolerance during the seedling stage are of great significance for stabilizing rice production. In this study, rice accessions XB and 19H19 were used to dissect the mechanisms underlying cold tolerance using RNA-seq, BSA-seq and genetic linkage analyses. RNA-seq analysis was performed on 19H19 (cold resistant) and XB (cold sensitive) seedlings treated with RT/LT and then subjected to recovery to obtain DEGs related to cold tolerance. The number and functional categories of DEGs differed between 19H19 and XB, regardless of the temperature and subsequent recovery. The genes that were uniquely differentially expressed in 19H19 and XB were primarily enriched in functions related to cold tolerance regulation, such as ‘phosphorylation’ and ‘phosphotransferase activity, alcohol group as acceptor’ [28]. The genes that underlie the common cold stress response in both lines are primarily enriched in functions related to the ‘chloroplast thylakoid membrane’ and ‘photosynthetic membrane’ [29]. *qCTS8* and *qCTS12* were overlapped with the cold tolerance QTLs reported in our previous studies [27, 37]. Moreover, the intervals of the two QTLs were narrowed down to 784 kb and 702.5 kb, respectively. The *qCTS12* was confirmed by BSA-seq (Figs. 6 and 7). Based on these results, a total of 112 candidate genes were identified by BSA-seq and genetic linkage analysis. These candidate genes belonged to eight functional categories, six of which (‘oxidation-reduction process’, ‘kinase activity’, ‘transferase activity’, ‘ATP binding’, ‘catalytic activity’, and ‘response to stress’) are related to the regulation of cold tolerance in rice [22, 33, 38–41]. Additionally, the trend of expression of candidate genes related to ‘oxidation-reduction process’ and ‘response to stress’ differed between XB and 19H19 seedlings during the cold treatment and subsequent recovery. These results suggest that difference in the expression levels of candidate genes in these two functional categories between 19H19 and XB may be closely related to the difference in the cold resistance of these two genotypes.

### Chloroplast genes are likely involved in the regulation of cold tolerance in rice

The chlorophyll levels are considered as an important indicator for evaluating photosynthetic capacity. LT stress can significantly down-regulate the expression of genes related to chlorophyll biosynthesis, chlorophyll binding proteins, photoresponse core complexes and chloroplast precursors, thus obstructing chlorophyll biosynthesis and consequently decreasing chlorophyll content [40, 42]. In addition, cold stress greatly suppressed the contents of photosystem II (PSII) and photosystem I (PSI) proteins in rice seedlings, which might lead to lower values of maximum PSII yield (Fv/Fm), suggesting that chlorophyll levels are an important indicator of the cold tolerance in rice [42]. Generally, rice materials with tolerance to low temperatures retain a relatively high capacity for photosynthesis because they can enhance energy metabolism and effectively scavenge reactive oxygen species (ROS) to withstand the stress caused by low temperatures [42]. Rice varieties with higher chlorophyll content exhibit greater cold tolerance [43]. Similarly, it was found that the chlorophyll content in 19H19 is significantly higher than that in XB in the present study. And the chlorophyll content of XB seedlings decreased continuously during the cold treatment and subsequent recovery; however, the chlorophyll content of 19H19 seedlings increased after the 7-day recovery treatment (Fig. 1D). That is say, the increase in the chlorophyll content for 19H19 during cold stress and recovery treatment which allow the plant to maintain its characteristics of photosynthesis, therefore promoting its cold tolerance. Transcriptome analysis of cold-tolerant and cold-sensitive rice genotypes under cold stress revealed that genes associated with chloroplasts, chloroplast thylakoids, carbon fixation in photosynthetic organisms, and photosynthesis-related genes are differentially expressed in various cold tolerant rice genotypes [44–46]. It is in line with our findings. More chloroplast genes is downregulated in XB than in 19H19, which might explain the cold sensitivity of XB (Fig. 4A, C). However, strikingly, the upregulated genes unique to the R19H19LTvs19H19LT group were mainly enriched in the chloroplast-related pathway, indicating that the upregulated genes restore the growth of 19H19 seedlings after the recovery treatment (Fig. 5A, C). Furthermore, 32 candidate genes screened out by BSA-seq and QTL mapping analyses were found to interact or co-expressin relation to chloroplast-relatedor photosynthesis-related genes. These results suggest that chloroplast genes are involved in the response to cold stress.

### Comparison of candidate genes identified in this study with previous CTS-related QTLs/genes

Of the 112 candidate genes identified in this study, two genes (*LOC_Os06g10880* and *LOC_Os02g29150*) have been cloned [47, 48]. Among these two genes, *LOC_Os06g10880* (*OsbZIP46*) encodes a bZIP transcription factor, which acts as a positive regulator of abscisic acid signaling and drought tolerance in rice. Interestingly, the expression level of *LOC_Os06g10880* was down-regulated in 19H19 and XB seedlings after the cold treatment, suggesting that this gene responds to cold stress. Additionally, based on the physical positions of markers flanking CTS-related QTLs and genes, 55 out of 112 candidate genes were located in chromosomal regions harboring QTLs identified in DXWR [37, 49–51].

Based on the results of gene expression analysis, two types of gene expression profiles were taken into account. In the first profile type, the candidate genes related to ‘oxidation-reduction process’ were downregulated in 19H19 seedlings after cold stress but upregulated in XB. In the second profile type, some of the candidate genes related to ‘response to stress’ showed no significant change in expression in 19H19 after cold stress but were upregulated in XB seedlings (Fig. 8). The functions of these candidate genes should be investigated further.

Among these 55 genes, five were related to two important cold tolerance-related functions (‘oxidation-reduction process’ and ‘stress response’) [10, 27, 37, 48, 49, 52]. Above-mentioned genes showed consistent expression level trends in 19H19 and XB seedlings (Fig. 8). The only gene related to ‘response to stress’ (*LOC_Os12g18729*) is located in the *qCTS-12* [37] and *qCTS12* regions, and its expression pattern differed between 19H19 and XB seedlings during the recovery period (Fig. 8). As expected, the expression level of *LOC_Os12g18729* increased in 19H19 during recovery, but decreased constantly in XB during the cold treatment and subsequent recovery. Accordingly, dynamic changes in the expression level of *LOC_Os12g18729* showed that the transcript abundance of this gene returned to normal levels in 19H19 seedlings during recovery but not in XB seedlings. These results imply that the failure of XB to regain the normal level of *LOC_Os12g18729* transcripts contributes to its cold sensitivity (Fig. 10).

### *LOC_Os12g18729* potentially plays a pivotal role in the interaction/coexpression network to regulate cold tolerance in rice

Our results indicated that *LOC_Os12g18729* potentially plays a pivotal role in regulating cold tolerance in rice. Therefore, we analyzed the sequence of *LOC_Os12g18729* in 19H19 and XB. A 42-bp deletion was detected in the third exon of *LOC_Os12g18729 *in XB, and the LOD value for the 42 bp deletion in the entire population is 5.42, with a PVE of 32.47 (Table 3). The genotype of BC_5_F_2_ individuals with SP values lower than 15% was consistent with that of XB. The introgression of naturally occurring favorable variation from DXWR into *indica* rice cultivars could promote the genetic improvement of CST in rice. In the network, DEGs related to ‘oxidation-reduction process’, ‘response to stress’, and ‘protein phosphorylation’ showed interaction with *LOC_Os12g18729* (Fig. 11B). Additionally, genes related to ‘oxidation-reduction process’ and ‘protein phosphorylation’ are reportedly involved in the regulation of cold tolerance in rice [2, 4]. This evidence confirms that *LOC_Os12g18729* plays an important role in the interaction/coexpression network to regulate cold tolerance. Moreover, the knockout mutant of *LOC_Os12g18729* decreased cold tolerance in early rice seedling stage signifcantly compared with that of ‘Nipponbare’ (WT) (Fig. 12B). These suggest that *LOC_Os12g18729* plays important role in the interaction/coexpression network to regulate cold tolerance in rice.

## Conclusion

DXWR exhibits greater cold resistance than cultivated rice. However, the molecular mechanism of cold tolerance in DXWR remains uncertain. In the present study, a BC_5_F_2_ population and its parents XB and 19H19 were employed to identify the cold tolerance-related genes for the early seedling stage in rice using three strategies (RNA-seq, BSA-seq and genetic linkage analysis). The chilling-tolerant line 19H19 was developed by crossing DXWR with XB. A total of two QTLs and 112 candidate genes were identified based on BSA-seq and genetic linkage analysis. These candidate genes were divided into eight functional categories. Among these candidate genes, the expression level of *LOC_Os12g18729* decreased in the LT treatment but was restored and enhanced during the recovery treatment in 19H19; by contrast, recovery treatment weakened the expression level of *LOC_Os12g18729* in XB. Additionally, XB contained a 42-bp deletion in the third exon of *LOC_Os12g18729*, and the genotype of BC_5_F_2_ individuals with a survival percentage lower than 15% was consistent with that of XB. In the network, DEGs related to ‘oxidation-reduction process’, ‘response to stress’ and ‘protein phosphorylation’ interacted with *LOC_Os12g18729. *The knockout mutant of *LOC_Os12g18729* decreased cold tolerance in early rice seedling stage signifcantly compared with that of WT. These findings would provide good candidates for the gene cloning and breeding application of cold tolerance genes from DXWR.

## Methods

### Plant materials, growth, and treatment conditions

An interspecific backcross between XB and DXWR was made in 1998; XB (*O. sativa* L. ssp. *indica*) is the maintainer line of the first commercial super hybrid rice Xieyou9308 in China. Voucher specimens were deposited at the Jiangxi Provincial Crop Germplasm Resource Bank, Jiangxi Academy of Agricultural Sciences (voucher No. SDB02459). And DXWR is a common wild rice (*O. rufipogon*) that originated in Dongxiang County, Jiangxi Province, China. Voucher specimens were deposited at the Jiangxi Provincial Crop Germplasm Resource Bank, Jiangxi Academy of Agricultural Sciences (voucher No. SDY48-15) and was identified as by Professor Yeqing Xiao. Rice line 5339, a BIL selected from the BC_1_F_10_ population of the interspecific backcross XB//DXWR/XB [49], was backcrossed with the recurrent parent XB and selfed to generate BC_4_F_2_lines, and the cold-tolerant introgression line 19H19 (BC_4_F_2_) was identified by five rounds of selection for tolerance to 8 °C/5°C (day/night) temperature for 5 days at the seedling stage. Subsequently, 19H19 was backcrossed with XB to generate 120 BC_5_F_2_ lines. Three recombinant plants with sequential heterozygous segments covering the interval of RM3246-RM27956, were selected from the BC_5_F_2_ generation. They were selfed to produce three corresponding BC_5_F_3_ populations, respectively. The *LOC_Os12g18729* mutants *LOC_Os12g18729*-1 and *LOC_Os12g18729-*2 (Nipponbare background) were generted by CRISPR-Cas9 system [53]. All DNA constructs and PCR products were confrmed by equencing (Tsingke Biotech, Beijing). Seeds of 19H19, XB, BC_5_F_2,3_ lines and Nipponbare and mutants were stored at 45 °C for 48 h to break dormancy, and then incubated on water-saturated filter paper in the dark at 36 °C for 48 h to induce germination. A total of 30 germinated seeds of each line were cultured in complete medium under natural conditions, with natural sunlight, until reaching the two-leaf stage. Then, rice seedlings were treated with LT (8 °C day/5°C night) for 4 d under 14-h light/10-h dark cycle, 20,000 lx light intensity, and 60% relative humidity. After the LT treatment, rice seedlings were allowed to recover at natural conditions for 7 d. The SP value of seedlings was calculated using the following equation [40]:

$$SP=\frac{Number of surviving seedlings}{Number of total seedlings}\times 100$$

The experiment was conducted using a randomized complete block design with three replications. BC_5_F_2_ individuals with SP ≥ 80% were considered to exhibit high cold tolerance, while those with SP ≤ 15% were considered to show low cold tolerance. These individuals were selected to establish the H- and L-bulks, respectively.

### Determination of cold stress-induced changes in leaf chlorophyll content

The chlorophyll content of leaves was estimated by ethanol soaking. Briefly, 500 mg of leaves were placed in a graduated tube containing 50 ml of 95% ethanol, and incubated in the dark for 36 h. The extract was filtered through a gauze to remove leaf debris. Colorimetry was performed at wavelengths of 665, 649, and 470 nm using a UV-Vis spectrophotometer (SHIMADZU U-V-1800PC, Japan). Finally, chlorophyll content (mg/g^− 1^) was calculated based on OD values using the equations proposed by Li et al. [54].

### RNA-Seq analysis

**Fig. 13:**
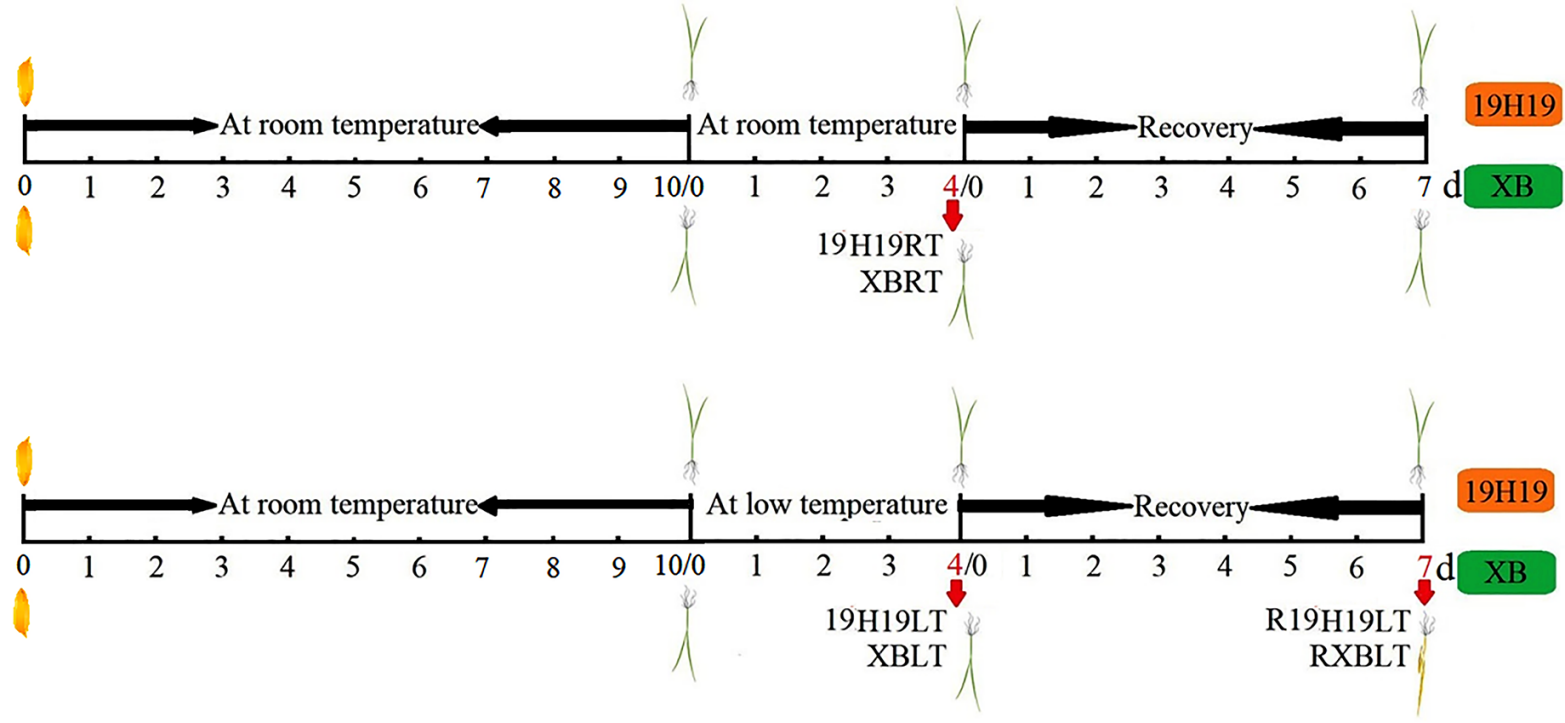
Schematic diagram showing the strategy of setting timepoint for sample collecting for the RNA-seq analysis of XB and 19H19

To study the dynamic changes in the transcript levels of rice genes during the cold stress and recovery treatments, the XB (cold sensitive) and 19H19 (cold resistant) seedlings were subjected to 4-d RT and LT treatments and a 7-d recovery treatment after the LT treatment (Fig. 13). Total RNA was extracted from whole seedlings, as described previously [55]. High-quality RNA was processed for library construction as described previously [56]. To ensure that the cDNA library was of high quality, the cDNA concentration and insert size were examined using Qubit 2.0 and Agilent 2100. High-quality cDNA libraries were pooled based on the pre-designed target data volume, and then sequenced on the Illumina sequencing platform (Biomarker, Beijing, China), generating a large amount of high-quality raw read data. High-quality clean reads were obtained by filtering the raw reads, and then mapped on to a pre-defined reference genome, generating mapped data. The mapped data were then subjected to basicanalyses including gene expression quantification, alternative splicing analysis, novel gene prediction and gene structure optimization. Fragments per kilo base of transcript per million mapped reads (FPKM) were estimated to quantify gene expression levels [57]. DEGs were identified using DESeq Rpackage v.1.18.0 based on two thresholds: fold change (FC) ≥ 2, and false discovery rate (FDR) ≤ 0.01. GO and KEGG pathway enrichment [58, 59] analyses of DEGs were performed based on the GO database (*Oryza sativa* (IRGSP-1.0) and KEGG database (http://www.genome.jp/kegg), respectively, according to the method described in Huang et al. [60].

### Experiment and bioinformatics procedures for QTL analysis based on linkage map construction

GBS (Genotyping by sequencing) libraries were constructed using the two-enzyme modification of the original GBS protocol [61].Then using a DNA1000 chip following a second column-cleaning, and library quantification was performed using PicoGreen (Invitrogen, Carlsbad, CA). Pooled libraries were adjusted to 10 nmol and sequenced with PE125 on the novaseq6000 (Illumina, San Diego, CA). Raw reads would be processed to get high quality clean reads according to the method described in Guo et al. [20]. To identify SNPs, the Burrows-Wheeler Aligner (BWA) was used to align the clean reads from each sample against the reference genome [62].Variant calling was performed for all samples using the GATK’s Unified Genotyper. SNPs were filtered using GATK’s Variant Filtration with proper standards (-Window 4, -filter “QD < 2.0 || FS > 60.0 || MQ < 40.0 “,-G_filter “GQ < 20”) and those exhibiting segregation distortion or sequencing errors were discarded. In order to determine the physical positions of each SNP, the software tool ANNOVAR [63], was used to align and annotate SNPs or InDels. The SNP variants outside the sequencing depth range of 5-500 would be considered missing data. To overcome the false positive of SNPs genotype of the population, the sliding window (10 kb) approach was used to evaluate a group of consecutive SNPs for genotyping. Then, the qualified SNP bin markers were used to construct the genetic linkage map using JoinMap 4.1. The regression algorithm and Kosambi mapping function were used in marker distance calculation. A Perl SVG module was used to draw the linkage map. QTL analysis was conducted with composition-interval mapping algorithm using the R/QTL software [64]. To determine the threshold of logarithm of odds (LOD) scores, 1000 times permutation was conducted and the threshold was selected at 5% confidence level. QTLs LOD values larger than threshold were called, for which the QTL location was determined, including 2-LOD drop support intervals. The regional genes were annotated and analyzed via the database of RAP *(http://rapdb.dna.affrc.go.jp) and Ensembl (http://plants.ensembl.org/Oryza_sativa).

To validate these QTLs, a total of eighteen markers, including thirteen SSR markers and five InDel markers were obtained (Table S9). The SSR markers were selected from the Gramene database (http://www.gramene.org), and the InDel markers were designed based on the whole-genome re-sequencing data of XB and DXWR. Subsequently, six polymorphic markers between XB and DXWR were used to further narrow down the target interval. Three recombinant plants with sequential heterozygous segments in the interval of RM3246-RM27956, were selected from the BC_5_F_2_ generation. They were selfed to produce three corresponding BC_5_F_3_ populations, respectively (Fig. S2). For BC_5_F_3_ populations, QTL mapping was performed using the default setting of the BIP (QTL mapping in bi-parental populations) approach in IciMapping V4.2 [65].

### BSA-Seq analysis

DNA was extracted from the young leaves of H-bulks (20 individuals), L-bulks (20 individuals), and their parents (19H19 and XB) using the cetyltrimethylammonium bromide (CTAB) method [66]. Four DNA libraries (XB, 19H19, H-bulk and L-bulk) were constructed using the Illumina TruSeq DNA Sample Preparation Kit (Illumina Inc., San Diego, CA, USA). The DNA samples were randomly nicked into 350-bp fragments by ultrasonication, ligated with adapters, and purified. The DNA libraries were sequenced on Illumina HiSeqTM PE150 (Glbizzia, Beijing, China). Low-quality reads containing adapter sequences were filtered, and the clean reads thus obtained were mapped on to the Nipponbare reference genome (*Oryza*_*sativa*_IRGSP-1.0) using the BWA software [67]. SNPs and indels were filtered using the GATK Variant Filtration function, with proper standards (-Window 4, -filter “QD < 4.0 || FS > 60.0 || MQ < 40.0 “, -G_filter “GQ < 20”). All mutations were annotated for genomic regions using ANNOVAR. SNP/Indel-index (scale of short reads containing SNPs and indels, different from the reference genome) and Δ(SNP/Indel-index) (SNP/Indel-index difference between H-bulk and L-bulk) were calculated to identify candidate regions for cold tolerance-related QTLs [68]. Then, SNP/Indel-index and Δ(SNP/Indel-index) graphs were constructed as described previously [69]. For candidate polymorphic marker sites, extract the annotation results of ANNOVAR, and prioritize selecting the genes that cause stop loss or stop gain, nonsynonymous mutations, variable splicing or frame shift mutations as candidate genes [70].

### Network analysis

BMK Cloud (http://www.biocloud.com/), an online open platform, was used for the WGCNA of all DEGs.

To further predict interactions, the regulatory network of genes-of-interest identified by genetic linkage map, BSA-seq and RNA-seq was inferred using the MERLIN algorithm, as described previously [30]. The transcriptome data obtained in this study and those downloaded from the National Center for Biotechnology Information (NCBI) were used. The data matrix comprised 182 samples from 14 experiments. Finally, integrating MERLIN and WGCNA networks, a BSA profile candidate gene and DEG interaction/coexpression regulatory network was constructed using Cytoscape v3.7.1.

### RT-qPCR assay

To detect the expression of the genes-of-interest in transcriptome and BSA profiling, the same total RNA samples (as used above) were reversely transcribed using the Reverse Transcriptase M-MLV (RNase H-) (Takara). Then, RT-qPCR analysis was performed using the SYBR^®^*premixEx Taq*^™^ II (Tli RNase Plus) (Takara), with *Osactin* (*Os03g0718100*) as the internal reference.

Primers used for RT-qPCR are listed in Table S9.

### Verification of candidate regions

To verify the candidate regions identified by BSA-seq, InDel markers were designed using Premier 5.0 based on the differences in nucleotide sequence between 19H19 and XB. Polymorphic Indel markers were then used to perform PCR amplification of 19H19 and XB DNAs using a thermocycler (ThermoFisher, USA) under the following conditions: initial denaturation at 94 °C for 5 min, followed by 35 cycles of denaturation at 94 °C for 30 s, annealing at 56 °C for 30 s and extension at 72 °C for 30 s. All PCR products were confirmed by sequencing (Tsingke Biotech, Beijing). Validation of mapping for InDel markers were performed using the default setting of the BIP (QTL mapping in bi-parental populations) approach in IciMapping V4.2 [65].

### Electronic supplementary material

Below is the link to the electronic supplementary material.

Supplementary Material 1

Supplementary Material 2

Supplementary Material 3

Supplementary Material 4

Supplementary Material 5

## Data Availability

The raw RNA-seq data have been deposited in Sequence Read Archive (SRA) (https://www.ncbi.nlm.nih.gov/bioproject/PRJNA991102).

## Abbreviations

ABA: Abscisic acid
BILs: Backcross inbred lines
BSA-seq: Bulked segregant analysis-sequencing
bZIP: Basic region/leucine zipper
CTS: Cold tolerance at the early seedling stage
DEG: Differentially expressed gene
DXWR: Dongxiang wild rice
FC: Fold change
FDR: False discovery rate
FPKM: Fragments per kilobase of transcript permillion mapped reads
GBS: Genotyping by sequencing
GO: Gene Ontology
H- bulks: High cold tolerance individuals
InDels: Insertion/deletion
KEGG: Kyoto Encyclopedia of Genes and Genomes
L-bulks: Low cold tolerance individuals
LT: Low temperature
MERLIN: Modular regulatory network learning with per gene information
NGS: Next generation sequencing
PS: Photosystem
PSR: Plant survival rate
PVE: Phenotypic variance explained
QTL: Quantitative trait locus
ROS: Reactive oxygen species
RT: Low temperature
RT-qPCR: Real-time quantitative polymerase chain reaction
SNPs: Single nucleotide polymorphisms
SP: Seedling survival percentage
WGCNA: Weighted gene co-expression network analysis
XB: Xieqingzao B
