# Supplementary figures and images for: Identification of candidate genes controlling cold tolerance at the early seedling stage from Dongxiang wild rice by QTL mapping, BSA-Seq and RNA-Seq
Source: BMC Plant Biol. 2024 Jul 9;24:649. doi: 10.1186/s12870-024-05369-x (PMC11232298; doi:10.1186/s12870-024-05369-x)

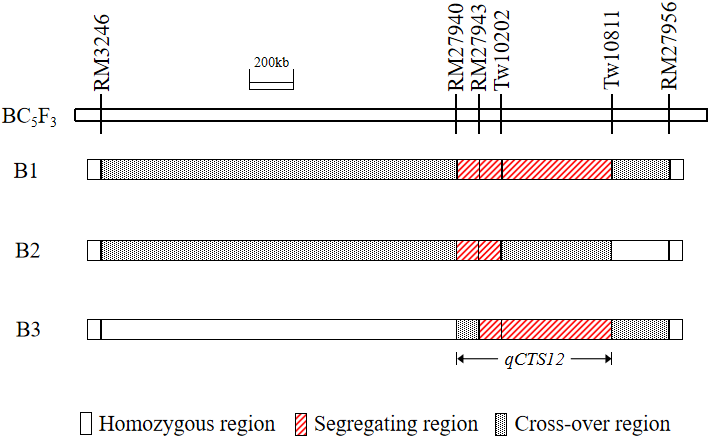


**Fig. S2.** Genotypic composition of three BC5F3 populations in the target region on chromosome 12.

Supplement: Supplementary file 1 — Supplementary Material 1 [file 12870_2024_5369_MOESM1_ESM.docx]

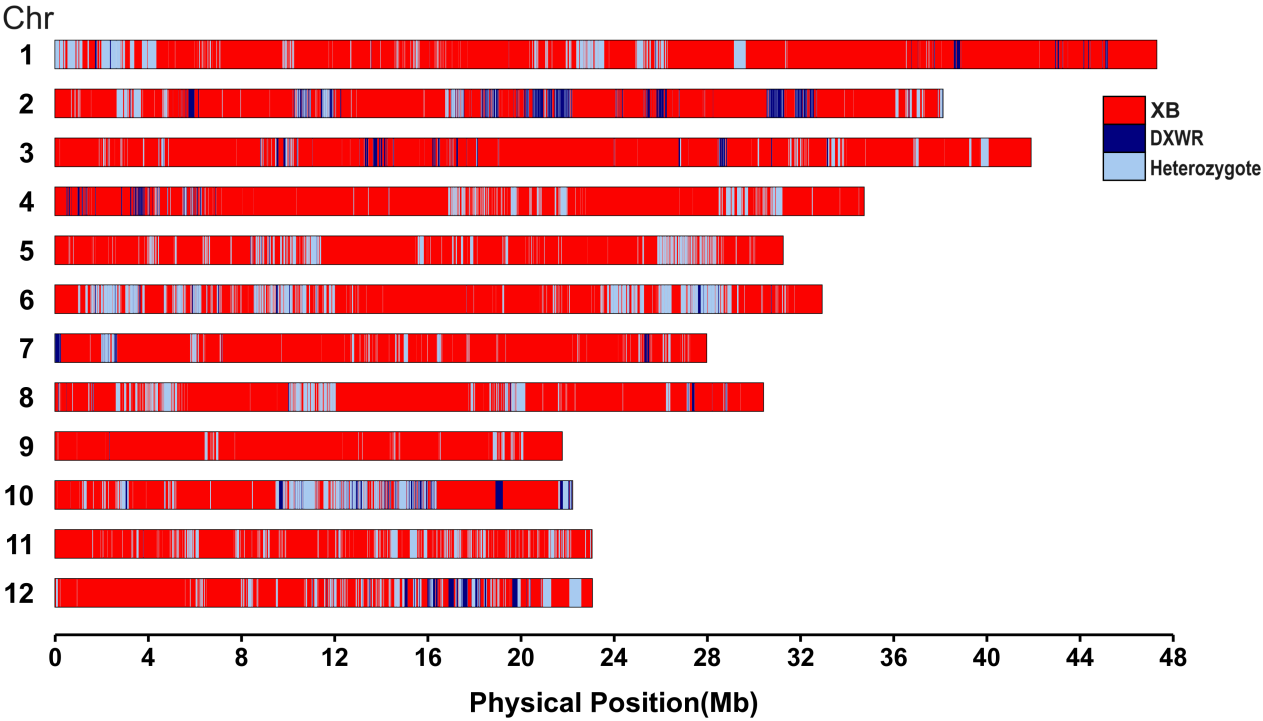


**Fig. S1.** Genotype of 19H19.

Supplement: Supplementary file 2 — Supplementary Material 2 [file 12870_2024_5369_MOESM2_ESM.docx]

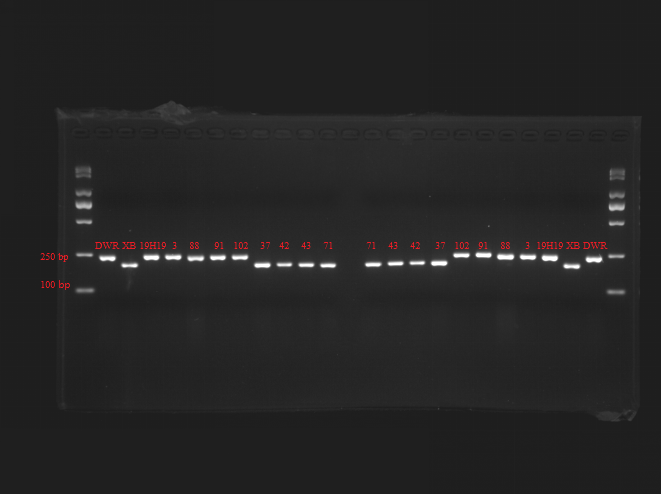


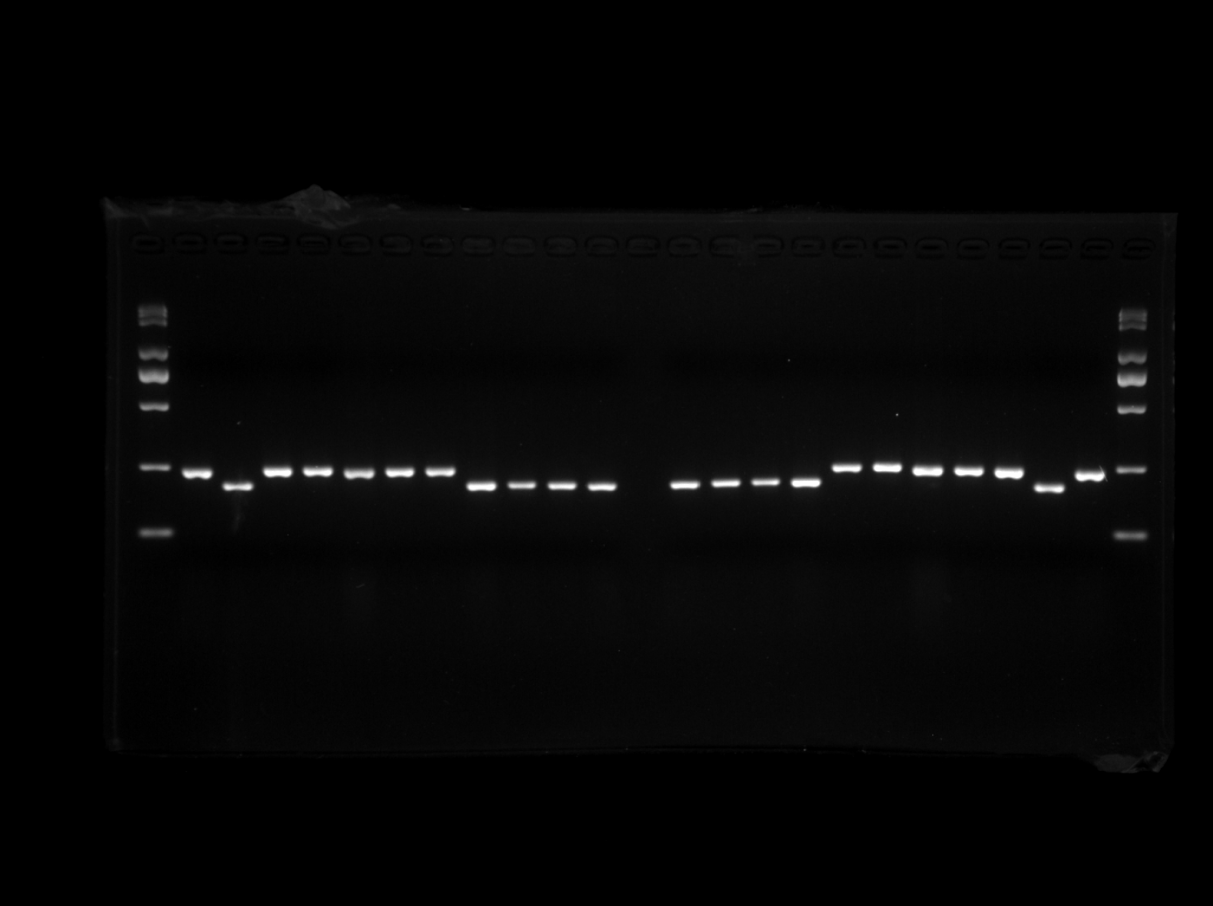


**Fig. S4.** The source data for Fig. 10.

| **SP values (B) of DXWR, XB, 19H19, and BC5F2 individuals （%）** | | | | | | | | | | |
| --- | --- | --- | --- | --- | --- | --- | --- | --- | --- | --- |
| DXWR | XB | 19H19 | 3 | 88 | 91 | 102 | 37 | 42 | 43 | 71 |
| 100 ± 1.5 | 5 ± 2.5 | 96 ± 2 | 98 ± 2 | 95 ± 1 | 98 ± 6 | 95 ± 1 | 5 ± 2 | 15 ± 1 | 0 | 1 ± 1 |

Supplement: Supplementary file 4 — Supplementary Material 4 [file 12870_2024_5369_MOESM4_ESM.docx]
