# Supplementary material for: Identification of candidate genes controlling cold tolerance at the early seedling stage from Dongxiang wild rice by QTL mapping, BSA-Seq and RNA-Seq
Source: BMC Plant Biol. 2024 Jul 9;24:649. doi: 10.1186/s12870-024-05369-x (PMC11232298; doi:10.1186/s12870-024-05369-x)
Supplement: Supplementary file 3 — Supplementary Material 3 [file 12870_2024_5369_MOESM3_ESM.doc]

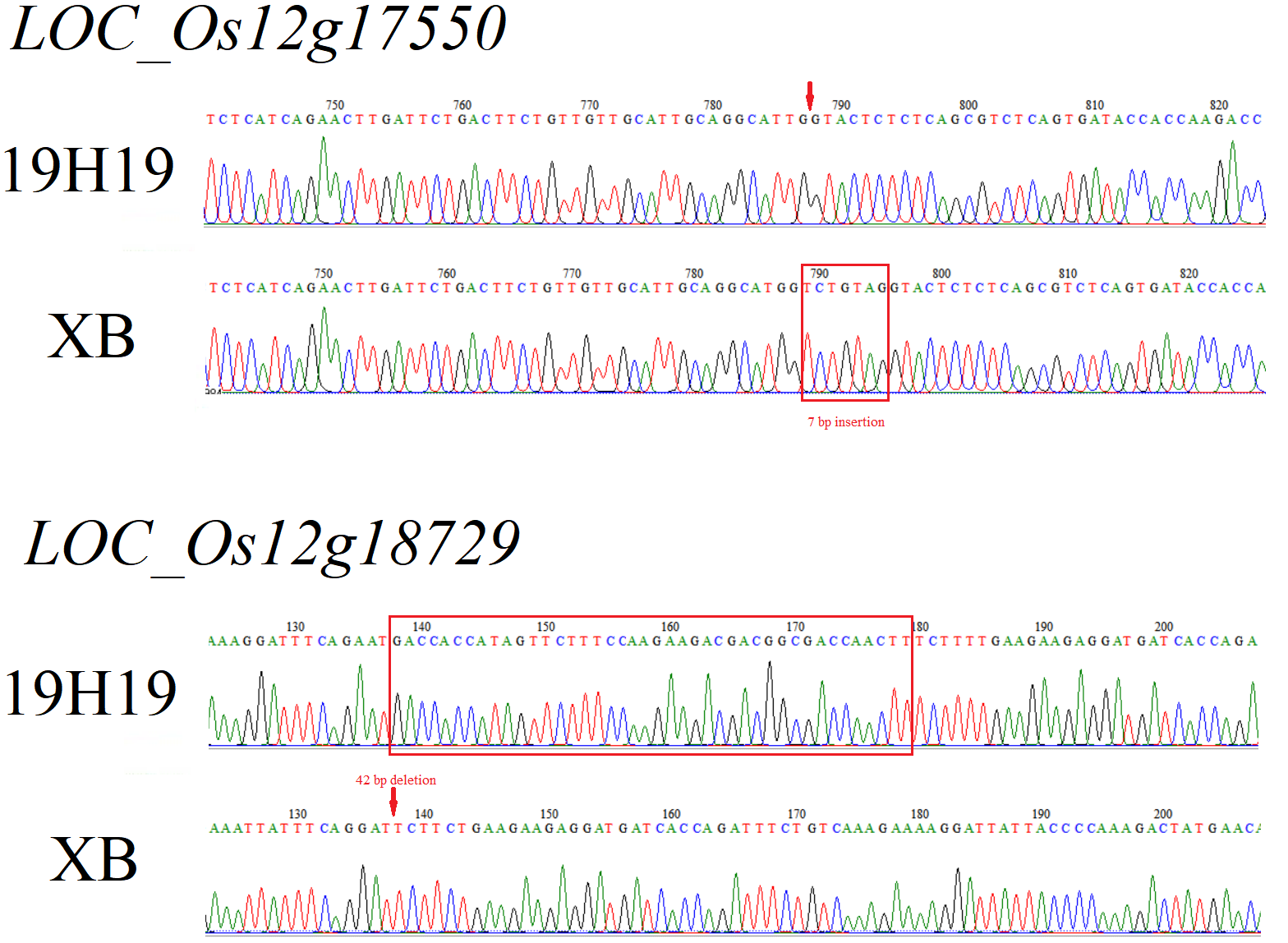


**Fig. S3.** The sequencing results of *LOC_Os12g17550* and *LOC_Os12g18729* in 19H19 and XB. Inserted nucleotide is indicated with red uppercase letter and red arrows represent the deleted bases.
